# Genetic variation and phylogeographic structure of the cotton aphid, *Aphis gossypii*, based on mitochondrial DNA and microsatellite markers

**DOI:** 10.1038/s41598-017-02105-4

**Published:** 2017-05-15

**Authors:** Xing-Ya Wang, Xian-Ming Yang, Bin Lu, Li-Hong Zhou, Kong-Ming Wu

**Affiliations:** 1 State Key Laboratory for Biology of Plant Diseases and Insect Pests, Institute of Plant Protection, Chinese Academy of Agricultural Sciences, Beijing, 100193 P. R. China; 2 Plant Protection College, Shenyang Agricultural University, Shenyang, Liaoning 110866 P.R. China; 3 Chengdu Institute of Biology, Chinese Academy of Sciences, Chengdu, Sichuan 610041 P.R. China; 4 Institute of Flower Research, Liaoning Academy of Agricultural Sciences, Shenyang, Liaoning 110161 P.R. China

## Abstract

*Aphis gossypii*, one of the most important agricultural pests in the world, can cause serious economic losses in the main crop-producing areas. To clarify issues such as the genetic differentiation, genetic structure, and demographic history of *A. gossypii* populations, we used 10 nuclear microsatellite loci (SSR) and two mitochondrial gene sequences (*COI* and *Cytb*) to investigate genetic diversity and population structure of *A. gossypii* populations that were collected from 33 sampling sites in China from different climatic zones. SSR and mtDNA data suggested low to moderate levels of genetic diversity. A star-shaped network of mtDNA haplotypes indicated that the maternal ancestor of China cotton aphids likely originated in Xinjiang. The POPTREE, STRUCTURE and principal coordinate analysis (PCoA) revealed two genetic clusters: an eastern and a western region group. Isolation by distance (IBD) results showed a positive correlation between geographic distance and genetic distance in the vast eastern region but not in the western region. Neutrality testing and mismatch distribution analysis provided strong evidence for a recent rapid expansion in most populations. Genetic bottleneck was not detected in *A. gossypii* populations of China. The present work can help us to develop strategies for managing this pest.

## Introduction

The cotton aphid, *Aphis gossypii* Glover (Hemiptera: Aphididae), is an important cosmopolitan pest that attacks numerous cultivated plants, such as Cucurbitaceae, Rutaceae and Malvaceae, causing serious economic losses in major crop areas around the world. Widely distributed in tropical, subtropical and temperate regions, it is the most common aphid and the primary target pest of cotton in the world^1^. Generally, adults and nymphs of this species feed on the underside of leaves or the growing tips of shoots, sucking juices from the plant, causing seedling death, leaf curl and withering, and serious yield loss. Aside from direct damage, this pest is an important vector of various viruses such as mosaic, crinkle, and rosette, and among others^2, 3^. *A. gossypii* is a highly fecund, extremely polyphagous species, and host-adapted races have been identified^2–6^. In general, this species can be holocyclic with *Hibiscus*, *Catalpa* or *Rhamnus* trees as primary hosts in China, the United States and Japan. Its life cycle includes both parthenogenetic and sexually reproductive phases^2^. In spring, each egg gives rise to a wingless viviparous, parthenogenetic female, followed by several more parthenogenetic generations during the spring and summer^7^. At the end of autumn, parthenogenetic females give rise to a single sexual generation of males and females, and mated females lay fertilized eggs, which represent the overwintering stage^2, 8^. To our knowledge, *A. gossypii* has developed resistance to all of the original major groups of synthetic insecticides in the last few decades and in virtually all cotton-growing areas worldwide^9–12^. Therefore, this pest has quickly become a major problem when chemical controls fail because of resistance. Thus, an effective control method is essential and urgent.

A better understanding of population genetic structure could help to manage aphid populations by providing more reliable estimates of population dynamics and the risk of resistance genes arising. The population genetic structure of an organism is determined by various factors, such as geographical barriers, ecological difference, and historical processes, as well as the dispersal ability of species^13^. In recent years, many molecular markers have been used to infer the phylogeny and biogeography of species to understand their patterns of evolution^14^. Owing to their locus-specific codominant, high abundance and high rates of transferability across species, microsatellite markers have been widely used in population genetics of aphid species^15–17^. Of course, cytochrome oxidase subunit I (*COI*) and cytochrome *b* (*Cytb*) genes also have a clear evolutionary pattern and evolve at a relatively moderate rate, which makes it suitable for reconstructing species and population-level phylogenies^18, 19^. Little information on the genetic variation and population genetic structure of *A. gossypii* has been available before the development of methods for identifying polymorphisms of microsatellites, which are now used for analyzing the population genetic structure of many organisms^20^. Previous studies have shown that genetic diversity of *A. gossypii* is structured by host plants, and its genetic diversity and host specialization are affected by natural selection pressure^21–23^. Distinct genetic differentiation was found in *A. gossypii* populations from its winter hosts and summer hosts^4, 6, 24, 25^. In North China, this species has two host-specialized biotypes (cotton and cucumber)^26^. By contrast, many studies have documented that genetic differentiation between localities was not significant, whereas genetic differentiation was high between host plant families. Low genetic diversity and a lack of significant differentiation have also been found in France^27, 28^. However, the studies had smaller sample sizes or a limited number of populations, which were insufficient for clearly assessing the genetic diversity and population structure of this species in different climatic zones^26–28^.

**Figure 1:**
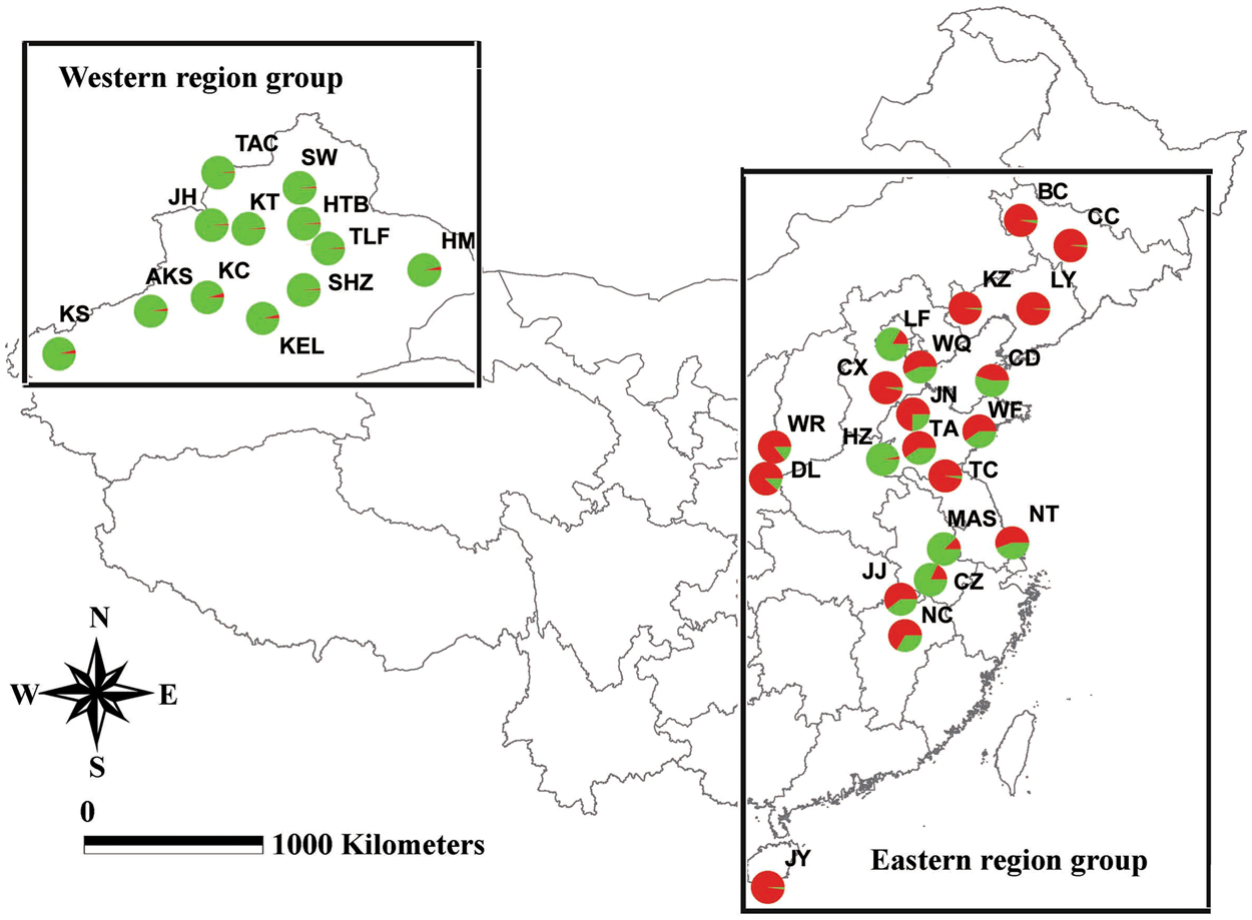
Sampling locations of *Aphis gossypii* and repartition of microsatellite lineages in greater China. Lineage 1 is shown in red and Lineage 2 is shown in green. Groups revealed by STRUCTURE analysis of microsatellite data are shown. For population abbreviations, see Table 2. ArcView GIS (version 3.2, http://www.resources.esri.com) was used to produce a distribution map based on the geographical coordinates of the localities, which were obtained with a Global Positioning System receive. The base map (Greater China 1: 4,000,000) for the analysis was obtained the URL: http://www.diva-gis.org/gdata.

Here, we examined the population genetics, including genetic variation, population structure and demographic history of 33 populations of *A. gossypii* in China (Fig. 1) using ten polymorphic nuclear microsatellite loci and two mitochondrial DNA sequence (*COI* and *Cytb*). We then discuss management strategies for this species based in the results, which also provide a theoretical framework for developing appropriate regional management strategies against this insect pest.

## Results

### Genetic diversity

In the present study, a total of 782 individuals from 33 populations were genotyped using ten microsatellite loci. Fisher’s exact tests showed that 111 of the 330 locus–population combinations deviated significantly from Hardy–Weinberg equilibrium (HWE). Significant linkage disequilibrium was present in 50 of 1485 tests (*a* = 0.05), and the studied loci were not linked with each other and were used in the subsequent analysis. The null allele frequency per locus was mostly less than 0.1 (Supplementary Table S1). The average *F*_ST_ not using the excluding null alleles (ENA) correction method was 0.224 and 0.213 using ENA per locus (Supplementary Table S2), and these *F*_ST_ estimations did not differ significantly (*P* = 0.4813). Therefore, the presence of null alleles had little influence on the estimation of *F*_ST_. The basic summary statistics of genetic variation among different geographic populations of *A. gossypii* in China based on the 10 microsatellite loci are presented in Supplementary Table S3. Overall, a low level of genetic diversity for the total populations in these study regions was obtained. The observed number of alleles (*N*_a_) across microsatellite loci ranged from 3.900 in Tacheng (TAC) to 8.400 in Baicheng (BC) and Changchun (CC), and had a mean of 6.103. The effective number of alleles (*N*_e_) values across microsatellite loci ranged from 2.013 in Heze (HZ) to 4.129 in Taian (TA) and had a mean of 3.223. The mean observed heterozygosity (*H*_o_ = 0.512) was similar to the mean expected heterozygosity (*H*_e_ = 0.574) in the total populations. The observed heterozygosity ranged from 0.291 in Liaoyang (LY) to 0.697 in Jinan (JN), whereas the expected heterozygosity ranged from 0.388 in HZ to 0.681 in TC. The unbiased expected heterozygosity (uH_E_) ranged from 0.395 in HZ to 0.698 in TC. Shannon’s information index (*I*) values across loci ranged from 0.748 in HZ to 1.537 in BC. Allelic richness (*A*_R_) across loci was 3.735, ranging from 2.455 (HZ) to 4.615 (BC).

**Figure 2:**
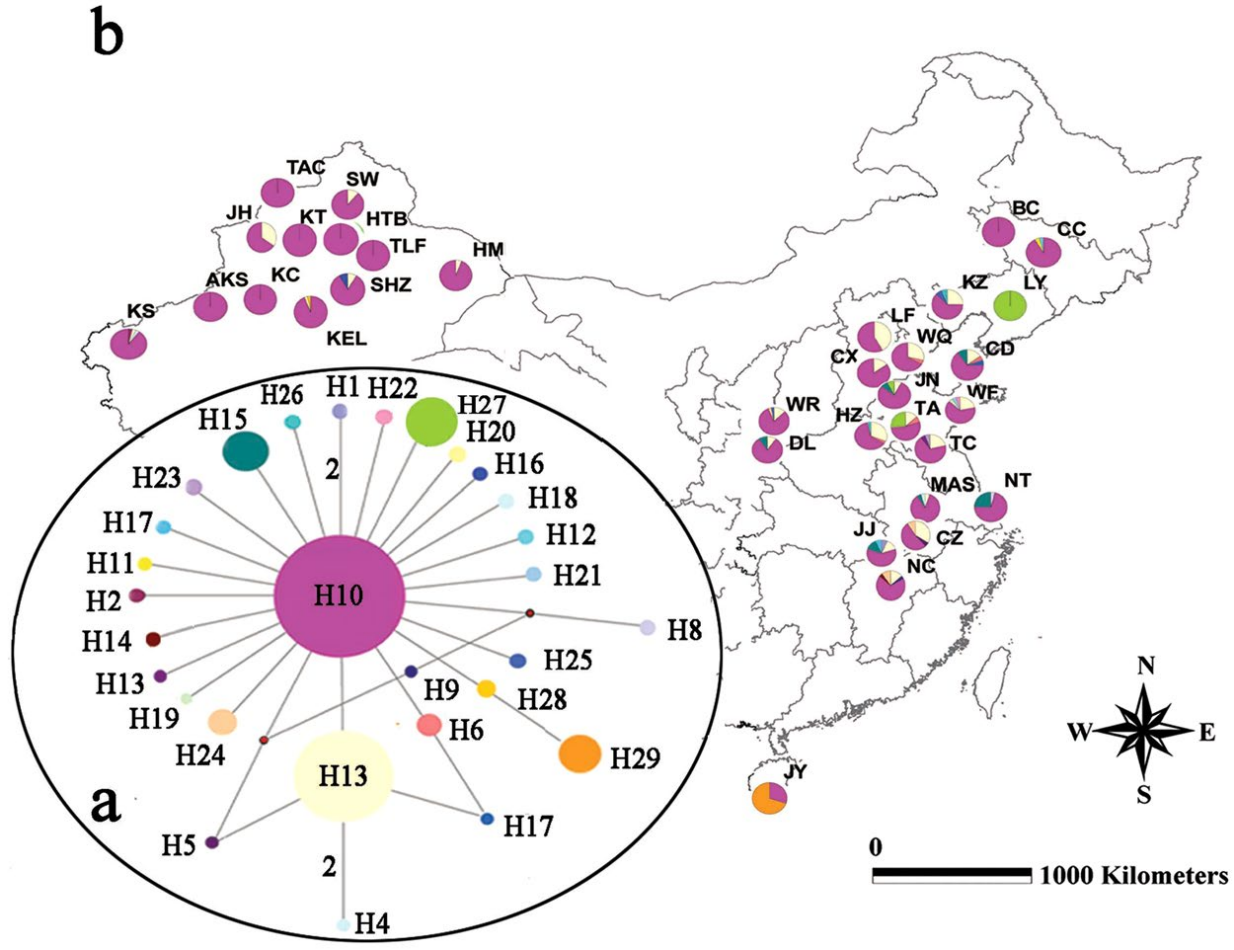
MtDNA haplotype network and haplotype frequencies in the examined populations of *Aphis gossypii*. (**a**) Haplotype network based on combined *COI* and *Cytb* sequences. Each haplotype was represented by a circle and identified by the codes H1–H29. The sizes of circles are proportional to the haplotype frequencies. Small red circles represent internal (unsampled) nodes. The median-joining network of the haplotypes of the combined *COI* and *Cytb* were generated by Network 2.0. (**b**) Haplotype frequencies and distribution in 33 populations. The color of each haplotype was the same as the network. For population abbreviations, see Table 2. ArcView GIS (version 3.2, http://www.resources.esri.com) was used to produce a distribution map based on the geographical coordinates of the localities. The base map (Greater China 1: 4,000,000) for the analysis was obtained the URL: http://www.diva-gis.org/gdata.

A region of 579 bases in the mtDNA *COI* genes from 782 individuals (deposited in GenBank under accession nos. KY069907–KY069921) and 629 bases of *Cytb* gene from 738 individuals (accession nos. KY069922–KY069936) were obtained. For subsequent analyses, we combined *COI* and *Cytb* and treated them as a single locus because of the lack of recombination in the mtDNA. Of the 1208 total characters, 1172 were conserved, and 36, that included 22 singleton polymorphic sites and 14 parsimonious informative sites, were variable. The distribution of haplotypes and genetic diversity among different populations of *Aphis gossypii* based on the combined *COI* and *Cytb* sequences was shown in Fig. 2a and Supplementary Table S4. Twenty-nine haplotypes were observed in 738 individuals. The average number of haplotypes in each population was 2.85 ± 1.39, ranging from 1 to 5. Weifang (WF), Changdao (CD), Jiujiang (JJ) and Nanchang (NC) had the highest number of haplotypes (5). In addition, H10 was the most common haplotype and shared by 551 samples and present in 32 of the 33 populations (except LY population). Thus, H10 is most likely to be the ancestral haplotype. The second most frequent haplotype (10.84%) was H3, which was shared by 80 individuals and present in 22 populations. Overall, the average Hd was 0.420, and the average Pi was 0.00062. The highest genetic diversity (Hd = 0.667, Pi = 0.00066) was detected in Taian (TA). However, in 5 localities among 3 provinces, including Baicheng (BC), Liaoyang (LY), Kuitun (KT), Tacheng (TAC) and Hutubi (HTB) genetic diversity was zero because no variable sites were found from these locations.

### Origin and distribution of mtDNA haplotypes

Phylogenetic analyses were conducted to determine the relationships among 29 *A. gossypii* haplotypes of the combined *COI* and *Cytb* and to detect any groups that can be explained by their geographic distribution. ModelTest indicated that the best substitution model was HKY + G. High congruence was observed between the phylogenies derived from maximum parsimony (MP) and maximum likelihood (ML) analysis, and these analyses generated only one inclusive group, but simultaneously provided many unresolved branches (Supplementary Fig. S1). These 29 mtDNA haplotypes were from 33 locations, which covered more than 4,000 km. These results reflected the random distribution of mtDNA haplotypes in *A. gossypii*, which may indicate a high level of gene flow in this species. Furthermore, the median-joining network of haplotypes showed that there was no apparent geographical clustering of mtDNA haplotypes (Fig. 2b). The haplotype network of combined genes obviously displayed a star-like pattern with the most common haplotype (H10) in the star’s center. H10 can be observed in all 6 cotton region of China (in 32 populations) and mainly distributed in Northwest China (Xinjiang Province), which raises the possibility that the maternal ancestor of China population was from Xinjiang. For most cases, only a 1-step mutation was found between the most common haplotype and the other haplotypes, which was often associated with demographic expansion.

### Population genetic differentiation

There was a moderate level of genetic differentiation among populations. Based on the microsatellite genotype data, pairwise *F*_ST_ values between the studied populations ranged from −0.04 to 0.58, and exact tests showed that 391 of the 561 pairwise populations differed significantly (Supplementary Table S5). For combined genes of *COI* and *Cytb*, pairwise *F*_ST_ values (ranging from –0.09 to 1.00) showed statistically significant genetic differentiation for 496 pairwise comparisons (*P* < 0.05) (Supplementary Table S6).

### Population genetic structure

Five methods were used to investigate the population structure and infer the relationships among *A. gossypii* populations.

### POPTREE analysis based on SSR

**Figure 3:**
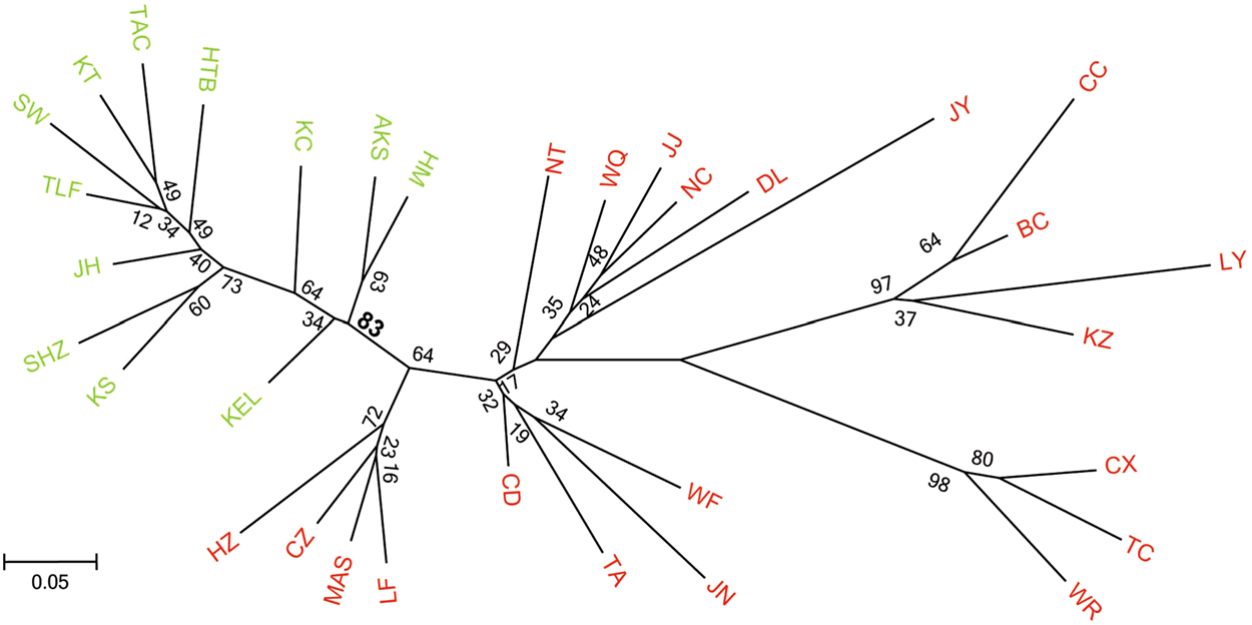
Unrooted NJ tree based on SSR data from 33 *Aphis gossypii* populations collected in China. Numbers beside the nodes are bootstrap values. Populations from Xinjiang Province, Northwest China with relatively stronger support, are marked as green, and the other populations, from eastern China, are marked as red.

The unrooted tree defined two major clades among 33 populations (Fig. 3). Populations from Xinjiang Province, Northwest China, including KT, TAC, TLF, JH, HTB, SW, KS, SHZ, KC, AKS, HM and KEL grouped together (bootstraps = 83). The other 21 populations from eastern China can be treated as one group because of the absence of a robust phylogeographic structure, which suggested frequent gene flow in the vast eastern region.

### Bayesian clustering

**Figure 4:**
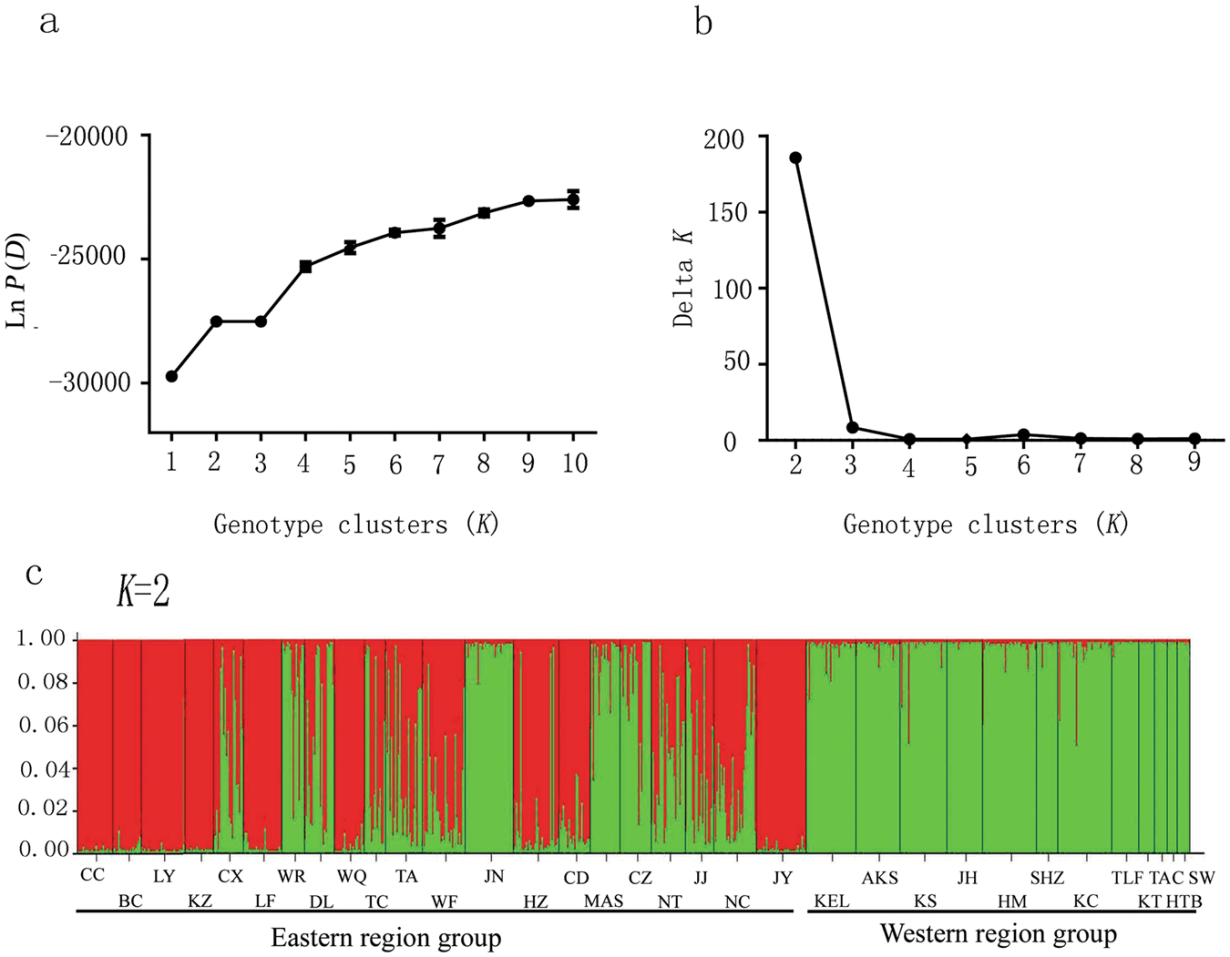
Bayesian inference to determine suitable cluster (*K*) and estimated cluster proportion using STRUCTURE for *Aphis gossypii*. The likelihood of the data given ln *P* (*D*) (**a**) and *ΔK* (**b**) are plotted against the number of genetic clusters (*K*). Error bars represent standard deviations over 10 runs. For the assignment proportion in all populations, each individual is represented by a thin vertical line, which was partitioned into *K* segments that represent its estimated population group membership fractions (**c**). Population codes are given in Table 2.

We used an admixture model implemented in STRUCTURE 2.3.3 software to explore different numbers of populations *K* to the population structure based on microsatellite data. STRUCTURE analyses produced stable solutions for *K* between 1 and 10, and the Δ*K* method determined that the best *K* was 2 according to the plateau criterion (Fig. 4), which was consistent with the hypothesis that these populations could be divided into two groups: eastern region group and western region group (Figs 1 and 4). Further structuring indicated the divergence of CC, BC, LY, KZ, LF, TC, and CD from the other eastern populations at *K* = 3 (Supplementary Fig. S2). A more fine-grained population structure was present at *K* = 4, and the subdivision still closely followed the geography. The Bayesian clustering method detected significant genetic clusters among the Northeast populations, with locations CC, BC, LY, and KZ in one cluster; LF, TC, CD, and DL in a second cluster, and the remaining populations, except Xinjiang, in a third cluster; the western populations (all from Xinjiang) grouped in a fourth cluster at *K* = 4 (Supplementary Fig. S2).

BayesAss analysis based on SSR data indicated that very limited and asymmetric gene flow existed between the eastern and the western region groups. Gene flow from west to east (0.0055) was greater than in the opposite direction (0.0016). Limited gene flow may be associated with moderate migration capability of the cotton aphid, habitat differences and lack of commercial trade between the east and the more isolated west, although potential dispersion routes (gene flow) from west to east are presented.

### Principal coordinate analysis (PCoA)

**Figure 5:**
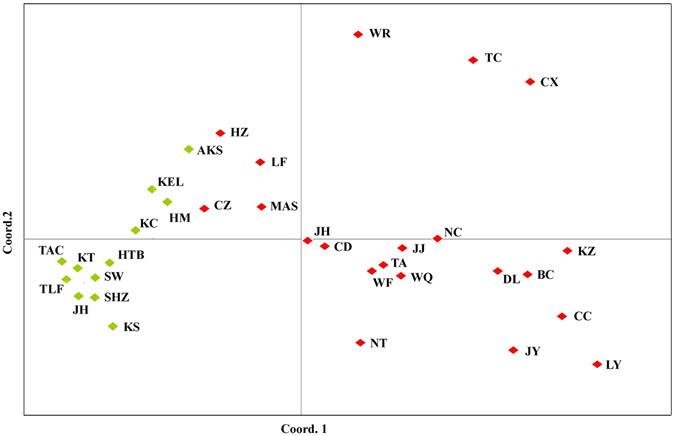
Principal coordinate analysis (PCoA) based on the genetic distance matrix of *F*_ST_ values for microsatellite data. Population codes are given in Table 2. Colors within the diamond: red, eastern region group; green, western region group.

The PCoA result (Fig. 5) showed a western–eastern pattern of genetic structure, which was similar with the POPTREE (Fig. 3) and STRUCTURE results (Fig. 4). The first and second axes explained 29.83% and 15.09% of the overall variance in microsatellite data, respectively (Fig. 5). Bayesian clustering based on data collected 782 individuals showed two distinct groups and supported the efficacy of the PCoA approach. We noticed that most of the geographically close populations resembled each other.

### Analysis of molecular variance (AMOVA)

Results of the AMOVA test on microsatellite and mtDNA markers in different populations and regional groups of *A. gossypii* are shown in Table 1. The global AMOVA of the data for the two molecular markers revealed that 23.16% (microsatellite) and 16.14% (mtDNA) genetic variation could be explained by the variation among populations, whereas the remaining came from variation within populations. Significant variability was found among microsatellites between the eastern and western groups (16.14%, *P* < 0.001) as inferred from above analyses. In contrast to SSR, mtDNA did not support the western–eastern pattern (−0.34%). Considering the limited variability (information) of the mtDNA here, which may due to the slower evolutionary rate of mtDNA and the especially rapid expansion of China cotton aphid populations (see the demographic results that follow), the AMOVA result from the SSR data showing moderate variability are more reasonable and can represent more recent events.

**Table 1:** Results of analysis of molecular variance (AMOVA) test on microsatellite and mtDNA markers in different populations of *Aphis gossypii* in China.

Molecular marker	Source of variation	Sum of squares	Variance components	Percentage variation (%)	*F*-statistics
Microsatellite	Global analysis				
	Among populations	370.35	0.22839 Va	23.26	*F*_IS_ = 0.086***
	Among individuals within populations	611.27	0.06492 Vb	6.61	*F*_ST_ = 0.233***
	Within individuals	537.00	0.68846 Vc	70.12	*F*_IT_ = 0.299***
	Total	1518.612	0.98177		
	Hierarchical AMOVA (*K* = 2)				
	Among groups	125.237	0.16467 Va	16.14	*F*_CT_ = 0.308***
	Among populations within groups	239.828	0.15011 Vb	14.71	*F*_SC_ = 0.175***
	Within populations	1077.689	0.70576 Vc	69.16	*F*_IS_ = 0.161
Combined *COI* and *Cytb*	Global analysis				
	Among populations	119.253	0.15742 Va	41.62	*F*_ST_ = 0.416***
	Within populations	155.666	0.22080 Vb	58.38	
	Total	274.919	0.37822		
	Hierarchical AMOVA (*K* = 2)				
	Among groups	3.915	−0.00128 Va	−0.34	*F*_SC_ = 0.417***
	Among populations within groups	115.338	0.15802 Vb	41.86	*F*_ST_ = 0.415***
	Within populations	155.666	0.22080 Vc	58.48	*F*_CT_ = −0.003***

***P < 0.001. Two groups including eastern region group and western region group based on STRUCTURE analysis (See Fig. 4).

### Mantel test for isolation by distance (IBD)

**Figure 6:**
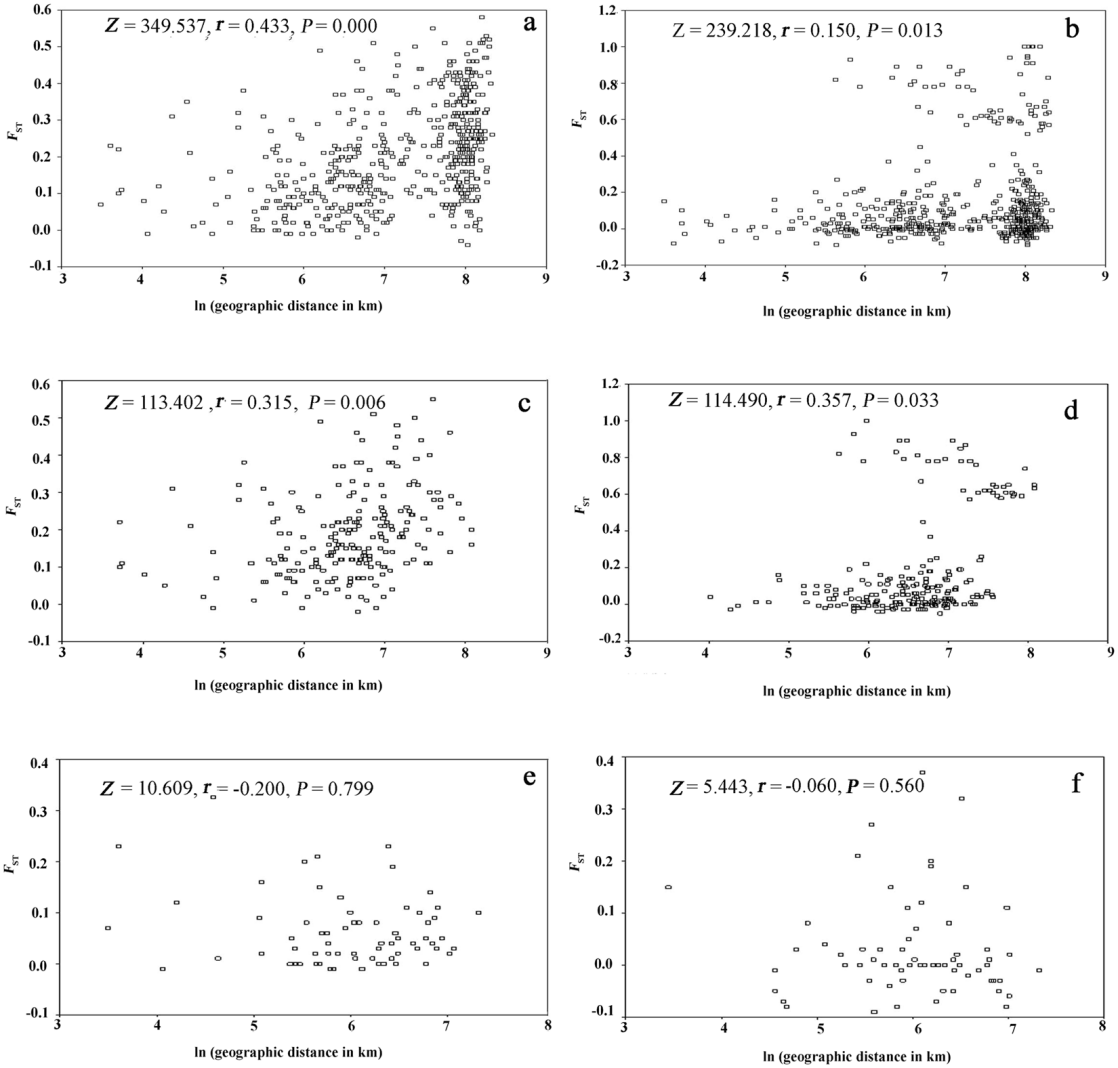
Correlation analysis between pairwise linearized *F*_ST_ values and the logarithm of geographic distance in Chinese populations of *Aphis gossypii* based on microsatellites ((**a**) total population; (**b**) eastern region group; (**c**) western region group) and combined *COI* and *Cytb* ((**d**) total population; (**e**) eastern region group; (**f**) western region group).

A Mantel test for IBD was performed to assess the correlation between the genetic distance matrix and the corresponding geographic distance matrix of *A. gossypii*. If the dispersal of *A. gossypii* is limited by distance, genetic and geographical distances should be positively correlated, producing a pattern of isolation by distance. Applying the Mantel test, significant positive relationships between genetic and geographic distances were found over the 33 populations of China cotton aphids based on both kinds of markers (microsatellite genotypes: *Z* = 349.537, *r* = 0.433, *P* = 0.000, Fig. 6a; combined *COI* and *Cytb*: *Z* = 239.218, *r* = 0.150, *P* = 0.013, Fig. 6b). Given that the isolated western and the eastern regions are far from each other and lack of gene flow, to minimize the distance influence, we performed separate IBD analyses for the western and the eastern group. Interesting, an IBD pattern was detected in the eastern region group of 21 populations (microsatellite genotypes: *Z* = 113.402, *r* = 0.315, *P* = 0.006, Fig. 6c; the combined genes of *COI* and *Cytb*: *Z* = 114.490, *r* = 0.357, *P* = 0.033, Fig. 6d) rather than in the western region group of 12 populations (microsatellite genotypes: Z = 10.609, *r* = −0.200, *P* = 0.799, Fig. 6e; combined *COI* and *Cytb*: *Z* = 5.443, r = −0.060, *P* = 0.560, Fig. 6f). The results were consistent with the moderate migration capability of cotton aphids. For the western region, the geographical scope is not large, and the simple habitat also makes migration feasible. Compared with the mtDNA data, the SSR data was more in line with the IBD pattern, perhaps as a result of the limited variability (information) of the mtDNA here, which may again be due to the slower evolution rate of mtDNA and especially rapid expansion of China cotton aphid populations.

### Demographic history analysis

The cotton aphid populations in China appear to have expanded demographically. Widespread single haplotype (Hap10) and classic “Star” shape of mtDNA haplotype network provide preliminary but strong evidence of expansion (Fig. 2). In addition, Tajima’s *D* ^29^ and Fu’s *F*_S_^30^ were calculated from the total number of segregating sites and used to assess the evidence for population expansion. For most populations in China, Tajima’s *D* value was significantly negative (Supplementary Table S4). When the 33 populations were considered as one or two groups, significantly negative values for combined *COI* and *Cytb* were obtained (total population: Tajima’s *D* = −2.218, *P* < 0.05, Fu’s *F*_S_ = −2.805; eastern region group: Tajima’s *D* = −2.014, *P* < 0.05, Fu’s *F*_S_ = −18,678; western region group: Tajima’s *D* = −1.764, *P* < 0.05, Fu’s *F*_S_ = −5.964). Furthermore, the unimodal mismatch distribution of all populations and the eastern region group and western region group indicated a sudden demographic expansion (Supplementary Fig. S3). The bottleneck analysis based on the microsatellite data, no significant heterozygote excess in 31 of the 33 populations (exceptions: HTB and SW) under the infinite allele model (IAM), two-phase model (TPM), and the strict stepwise mutation model (SMM). Meanwhile, these results were consistent with a normal L-shaped distribution of allelic frequency, indicating that *A. gossypii* had expanded demographically without a severe bottleneck in most regions of China (Supplementary Table S7).

It is more likely that the mitochondrial loci reflect the patterns of resistance of other genes and are thus a by product of selection. When we scanned for signals of selection, the *Z*-tests of selection rejected the null hypothesis of strict-neutrality (dN = dS) in favour of purifying selection (dN < dS; *P* = 0.002) rather than for positive selection (dN > dS; *P* = 1.000).

## Discussion

### Genetic diversity

Deviations from HWE were observed at multiple loci in multiple populations. Fisher’s exact tests showed that 111 of the 330 locus/population combinations deviated significantly from HWE. The specific reproductive mode of parthenogenesis might also result in deviation from HWE among the studied populations. In general, aphid population genetic studies have more often documented populations not in HWE because of heterozygote deficits (i.e., homozygote excess)^15, 31^, presumably because of clonal selection. Permanent parthenogenetic clones have high heterozygosity as a consequence of accumulated mutations^31–34^. The *F*_IS_ values of the 10 microsatellite loci evaluated in this study were a little less than zero, which indicated a deficit of heterozygotes (Supplementary Table S3), which in turn reflected a balance in the asexual ratio of the population. Overall, the average expected heterozygosity (*H*_e_) was similiar to the observed (*H*_o_). Note that a low *H*_o_ was obtained in some populations such as Liaoyang (*H*_o_ = 0.291), which suggested a high genetic identity within the population. These results could be attributed to the specificity of the geography and climate in Liaoning in northeastern China because aphids usually reproduce sexually at relatively high latitudes and low temperatures.

Genetic variability is fundamental for adaptive processes in a species. The genetic diversity found in *A*. *gossypii* through the use of molecular markers has shown that the clonal diversity is structured by its host plant^34, 35^. In the present study, *A*. *gossypii* had low to moderate genetic diversity based on microsatellite (Supplementary Table S3) and mitochondrial DNA data (Supplementary Table S4), and *H*_e_ varied between 0.388 (in HZ) and 0.681 (in TC). Similar cases of moderate genetic diversity have been documented in this species; *H*_e_ ranged from 0.409 to 0.873 on the basis of eight microsatellite loci^17^. Within a glasshouse population of *A. gossypii*, clonal diversity declined significantly as the spring/summer season progressed^22^. Low levels of genetic diversity were also found previously for other aphid species such as *Eriosoma lanigerum* ^36^ and *Myzus persicae* ^37^, which resulted from the adaptation of the aphids to two heavy selection pressures, the distribution of host plants and the use of insecticides^22, 27^. In general, a critical feature of aphid biology is their viviparous reproduction by cyclical parthenogenesis, which can result in numerous overlapping generations in a short period and an extremely rapid increase in population size, which could explain the low level of genetic variability detected in aphids as compared with other insects^7^. Additionally, aphids are also very sensitive to selection. *A. gossypii* is a particularly polyphagous species; it has been described on almost 300 host plants from various botanical families^3^. Because the eastern populations had more genetic diversity than in the western populations (Supplementary Table S4; eastern: Hd = 0.549; Pi = 0.00086; western: Hd = 0.141; Pi = 0.00014), fragmented habitats, migration between populations and genetic drift probably contributed to the loss of diversity in the western populations.

### Single origin and genetic structure

Compared with conventional phylogenetic trees, haplotype networks based on the combined *COI* and *Cytb* provide an enhanced view of the relationships among haplotypes and are preferable for intraspecific analyses because ancestral haplotypes may still be extant^38^. H10 was the most common haplotype of mtDNA and shared by 551 samples and present in 32 of the 33 populations (Fig. 2). Combined with the classic “star” shape of the network, the results indicated that these China cotton aphid populations arose from one or only a few maternal ancestors in Xinjiang and then quickly spread geographically. The mtDNA tree and network did not reveal any distinct geographic distribution pattern for this pest (Fig. 2), which may be due to the relatively slow evolutionary rate of the aphid and the “maternal inheritance” pattern of this maker. In contrast, microsatellite DNA shows more variation information than mitochondrial gene, which may reflect more recent events, including population divergence, gene flow and bottleneck. The China cotton aphids separated into two genetic groups as supported by the PCoA, POPTREE, STRUCTURE analysis (Figs 3, 4 and 5) and AMOVA result (Table 1). Beside the fact that the ancestor haplotype (H10) of mtDNA from Xinjiang was widely distributed in eastern populations, we also detected limited and asymmetric nuclear gene flow from the western to eastern region (0.0055), indicated that the eastern populations migrated from the western region over time. Geographic barriers and climatic factors might contribute to the isolation of the studied population and play an important role in preventing gene flow among groups^39, 40^; for example, mountains shaped the population structure of the terrestrial species *Locusta migratoria* ^41^ and *Paeonia rockii* ^42^. Therefore, we speculate that the differing climates and large mountain systems (Qilianshan, Qinling, and Taihang Mountains) in the western and eastern region might have prevented or slowed nuclear gene flow between the eastern and western populations, resulting in lineage differentiation. Furthermore, for the JY population in south China (Jiyang, Hainan province), there is one mtDNA haplotype (H29) which was more divergent from H10 (Fig. 2). H29 were found in 23 of the 33 individuals in JY population. Taking into account the larger geographical distance between JY and other populations, distance may also play a role in population differentiation of China cotton aphid.

Moderate flight and dispersal ability of the aphids might allow short-distance movement. Usually, IBD effects are pronounced in moderately mobile species but weak in both low- and high-mobility species, since extensive gene flow homogenizes populations across large geographic areas^43^. Therefore, we have reason to assume that there is a positive correlation between genetic and geographic distances among the populations. However, an IBD pattern was detected in the eastern region, not in the western. For the western region, geographical scope is not large, and the simple habitat and climate also make migration and gene flow feasible. For the vast eastern region, air-flow direction and monsoons can affect long-distance migration behavior though the difference in climate from north to south is great. Human-mediated dispersal of this pest might be an important mode of spread along the eastern coastline of China, perhaps through the transport of seedlings. Pairwise nuclear *F*_ST_ between populations of JY (Hainan Province) and Northeast China (CC, BC, LY, KZ) was not large though the two sites are separated by more than 3000 km (Fig. 1). Considering the insect’s moderate migration ability, commercial transport may be responsible. For this reason, lack of commercial trades as well as geographic barriers between the east and the more isolated west likely decreased gene exchange between the two regions. That is also an important reason for our defining all the populations in the eastern region as one genetic group. Generally speaking, IBD still played a major role in genetic differentiation in the eastern region though the factors already mentioned also have an effect.

### Pest management implications and future work

Increasing our understanding of the population dynamics of *A. gossypii* may improve predictions of its outbreaks and enhance management efforts. The present work provided robust evidence of population expansion. The widespread occurrence of single mtDNA haplotype (H10), which was detected in all 33 sampled populations, is the strongest evidence of expansion. The large negative values of Tajima’s *D* and Fu’s *F*_S_ statistic and the unimodal mismatch distribution are also evidence of population expansion in most populations of cotton aphids in China. SSR data indicated that these cotton aphids experienced a recent demographic expansion without a severe bottleneck in most regions in China. As noted already, the results will provide essential information for understanding possible local adaptation and dispersal patterns and for further clarifying the relationship between genetic variation and outbreaks of *A. gossypii*. In the future, we will focus on monitoring the population dynamics of this species and understanding its seasonal occurrence in the main crop-producing areas of China to provide more useful data for forecasting outbreaks and managing this species.

Over the past several decades, pest management programs, such as cultural controls, biological controls, and the use of insecticides have been used in China and have partially prevented the damage and spread of *A. gossypii*. Unfortunately, *A. gossypii* has developed resistance to insecticides such as carbamate and organophosphates^9, 44–46^, and the exponential growth associated with parthenogenesis in this aphid species favours rapid selection for insecticide resistance mechanisms as a consequence of intense insecticide use^9, 11, 12^. It is more likely that the mitochondrial loci reflect the patterns of resistance of other genes and are thus a byproduct of natural selection. However, we did not detect any signal for positive selection on *COI* or *Cytb*. Positive selection may act on detoxification enzymes and metabolic genes like P450 family. High gene flow among the cotton aphid populations may explain the rapid spread of insecticide-resistance genes, and many resistance characteristics also accumulate through long-distance migration. Therefore, to survey for populations of *A. gossypii* with insecticide resistance over large temporal and spatial scales and investigate the distribution of resistance genes relative to the genetic structure and level of gene flow among populations of *A. gossypii* in China, quantifying the level of resistance to insecticides will be important for devising application strategies for chemical pesticides. In addition to geography and climate, host plant specialization and insecticide treatment can also affect the distribution of genetic variation in aphid populations^27^.

The study of the temporal dynamics of genotypic diversity of *A. gossypii* populations in the cotton-growing area in Xinjiang over several consecutive years would help to reveal the relationship between the low genetic diversity and the adaptation of the aphids to two heavy selection pressures, the distribution of host plants and the use of insecticides. Additionally, we also found evidence of differentiation between the Xiuyan population, which was collected from *Rhamnus koraiensis*, and other populations collected from cotton (Wang *et al*. unpublished). Obviously, a broad-scale sampling strategy on different host plant families is needed. Therefore, future work should concentrate on the genetic differentiation between winter hosts and summer hosts through extensive sampling in China. Simultaneously, we will also focus on the relationship of the incidence of aphid populations on cotton, natural selection pressure (e.g., host rotation, host resources and pesticides), and genetic structure. Our genetic data suggest significant genetic differentiation among different strains of *Wolbachia* symbiosis, which is present between the eastern region and western region (Wang *et al*. unpublished). Therefore, future experiments should shed light on the influence of the bacterium on the diversity and genetic structure of cotton aphid populations. Developing additional microsatellite markers and more extensive sampling, including samples at a finer distance scale, should improve the resolution of the understood population genetic structure and our understanding of intra- and inter-race diversity of this species.

## Methods

### Sample collection and DNA extraction

All individuals of *A. gossypii* were collected from 12 provinces at 33 geographic locations in China, covering four climatic regions—mid-temperature zone (MTZ), warm temperate zone (WTZ), tropical zone (TZ), and subtropical zone (SZ)—from June 2012 to December 2015 (Table 2; Fig. 1). Samples in Changdao were collected with a suction trap (Keyun ST-1B), which was developed by the Institute of Zoology, Chinese Academy of Sciences. For preventing over-representation of siblings from each locality, each aphid was collected at least 1 m from the next sample. All the samples were preserved and stored at −20 °C in 95% ethanol and kept at the Plant Protection Institute, Chinese Academy of Agriculture Sciences (Beijing). Total DNA was extracted separately from each *A. gossypii* using a DNeasy extraction kit (Qiagen, Germantown, MD, USA) according to the manufacturer’s protocols *Aphis glycines* (GenBank accession nos. JQ860254 and GU205350) and *Aphis craccivora* (GenBank accession nos. AB506714 and AM085376) were used as outgroup species in the phylogenetic analysis.

**Table 2:** Habitat location, sample size and regional group information of *Aphis gossypii* in different geographic populations.

Geographic region	Site code	Site/Climatic zones	Latitude/Longitude	Collection date	Altitude (m asl)	Group
Northeast China	CC	1 Changchun, Jilin/MTZ	43.81 °N/125.41 °E	12 June 2012	219	E
	BC	2 Baicheng, Jilin/WTZ	44.82 °N/123.08 °E	12 July 2012	148	E
	LY	3 Liaoyang, Liaoning/WTZ	41.27 °N/123.23 °E	9 June 2012	27	E
	KZ	4 Kazuo, Liaoning/WTZ	41.29 °N/119.92 °E	10 July 2012	249	E
North China	CX	5 Cangxian, Hebei/WTZ	38.32 °N/116.60 °E	2 August 2012	35	E
	LF	6 Langfang, Hebei/WTZ	39.51 °N/116.58 °E	23 August 2012	39	E
	WR	7 Wanrong, Shanxi/WTZ	35.42 °N/110.56 °E	19 June 2012	491	E
	DL	8 Dali, Shannxi/WTZ	34.81 °N/110.11 °E	25 July 2013	334	E
	WQ	9 Wuqing, Tianjin/WTZ	39.08 °N/117.19 °E	31 August 2012	10	E
East China	TC	10 Tancheng, Shandong/WTZ	34.61 °N/118.37 °E	8 August 2012	148	E
	TA	11 Taian, Shandong/WTZ	36.18 °N/117.08 °E	10 August 2013	167	E
	WF	12 Weifang, Shandong/WTZ	36.38 °N/119.75 °E	10 August 2012	143	E
	JN	13 Jinan, Shandong/WTZ	36.68 °N/117.02 °E	11 August 2012	148	E
	HZ	14 Heze, Shandong/WTZ	35.26 °N/115.46 °E	12 August 2012	54	E
	CD	15 Changdao, Shandong/WTZ	38.40 °N/120.92 °E	27 August 2014	84	E
	MAS	16 Maanshan, Anhui/WTZ	31.70 °N/118.11 °E	21 August 2013	19	E
	CZ	17 Cizhou, Anhui/WTZ	30.66 °N/117.49 °E	22 August 2012	15	E
	NT	18 Nantong, Jiangsu/SZ	31.95 °N/121.31 °E	2 August 2012	2	E
Central China	JJ	19 Jiujiang, Jiangxi/SZ	29.71 °N/115.99 °E	10 July 2012	41	E
	NC	20 Nancang, Jiangxi/SZ	28.68 °N/115.89 °E	13 July 2012	16	E
South China	JY	21 Jiyang, Hainan/TZ	18.28 °N/109.58 °E	11 December 2015	15	E
Northwest China	KEL	22 Korla, Xinjiang/WTZ	41.73 °N/86.17 °E	July 2015	934	W
	AKS	23 Aksu, Xinjiang/WTZ	41.17 °N/80.26 °E	July 2015	1107	W
	KS	24 Kashi, Xinjiang/WTZ	39.47 °N/75.99 °E	July 2015	1298	W
	JH	25 Jinghe, Xinjiang/WTZ	44.60 °N/82.89 °E	July 2015	320	W
	HM	26 Hami, Xinjiang/WTZ	42.82 °N/93.51 °E	July 2015	759	W
	SHZ	27 Shihezi, Xinjiang/WTZ	44.31 °N/86.08 °E	July 2015	482	W
	KC	28 Kuche, Xinjiang/WTZ	41.72 °N/82.96 °E	July 2015	1071	W
	TLF	29 Tulufan, Xinjiang/WTZ	43.83 °N/87.61 °E	July 2015	900	W
	KT	30 Kuitun, Xinjiang/MTZ	44.42 °N/84.90 °E	July 2015	481	W
	TAC	31 Tacheng, Xinjiang/WTZ	46.75 °N/82.98 °E	July 2015	548	W
	HTB	32 Hutubi, Xinjiang//WTZ	44.01 °N/87.31 °E	July 2015	520	W
	SW	33 Shawan, Xinjiang/WTZ	44.87 °N/87.20 °E	July 2015	528	W

Sites are labeled according to abbreviated location names for *Aphis gossypii*. MTZ, mid-temperature zone; WTZ, warm temperate zone; TZ, tropical zone; and SZ, subtropical zone; E, eastern region group; W, western region group.

### Mitochondrial DNA amplification and sequencing

We sequenced two partial regions of the mitochondrial genes *COI* and *Cytb*. To amplify *COI*, we used published primers CIS (5′-ACCAGTTTTAGCAGGTGCTATTAC-3′) and CIA (5′-GTATATCGACGAGGTATACCATTT-3′)^47^ and for *Cytb*, CP1 (5′-GATGATGAAATTTTGGATC-3′) and CP2 (5′-CTAATGCAATAACTCCTCC-3′)^48^. Each PCR mixture contained 0.25 μL EasyTaq DNA polymerase (5 U/μL), 2.5 μL 10× Easy Taq buffer, 0.5 μL dNTP mixture (2.5 mM of each), 0.5 μL of each primer (10 pM), 1 μL of DNA template and 19.75 μL of distilled water, making a final volume of 25 μL. The reactions were performed in a GeneAmp PCR System 9700 (Perkin Elmer Applied BioSystems, Foster City, CA, USA) under the following conditions: 94 °C for 5 min; 35 cycles at 94 °C for 1 min, 52 °C for *COI* or 48 °C for *Cytb* for 1 min, 72 °C for 1 min; and a final extension at 72 °C for 10 min. All specimens were sequenced on an Applied Biosystems ABI 3730 DNA sequencer (Applied Biosystems). Chromatograms, including sense and antisense, were edited and assembled using DNASTAR 5.0 (DNASTAR, Madison, WI, USA) to obtain a single consensus sequences.

### Microsatellite amplification and genotyping

Ten microsatellite loci were used in this study using the methods of Michel *et al*.^49^ for loci *ago59* and *ago89*; Kim *et al*.^50^ for loci Agl2-6, AgI1-10, AgI1-11 and AgI1-2; and Gauffre & Coeur^51^ for loci AF-93, AF-45, AF-F and AF-beta, which were assigned unique fluorophores for fluorescent tagging of DNA in a PCR reaction. For these isolated microsatellite, each 20 mL reaction volume for the genotyping PCR contained 0.2 μL EasyTaq DNA polymerase (5 U/μL), 2.0 μL 10× Easy Taq buffer, 0.2 μL dNTP mixture (2.5 mM of each), 0.4 μL of reverse primers (10 pM), 0.4 μL of forward primer, which were labeled with fluorochromes (HEX or FAM) (10 pM), 0.5 μL of DNA template and 16.3 μL of distilled water. For the thermal profile, an initial denaturation step at 94 °C for 4 min was followed by 30 cycles of denaturing at 94 °C for 30 s, annealing at 58 °C for 30 s, and extension at 72 °C for 30 s; and a final 5 min extension at 72 °C. Following amplification, the products were visualized at Sangon Biotech Co., Ltd. (Shanghai, China) using an ABI 3730XL automated sequencer (Applied Biosystems). Microsatellite alleles were analyzed using GeneMapper 4.0 (Applied Biosystems).

## Data analysis

### Mitochondrial data

#### Genetic variation and diversity

The sequences were first aligned with CLUSTAL X 1.81^52^ using the multiple alignment default parameters and corrected by hand. Nucleotide composition, conserved sites, variable sites, parsimony informative sites were analyzed with MEGA 6^53^. Molecular diversity indices such as haplotype diversity (Hd) and nucleotide diversity (Pi) were estimated using DnaSP 4.0^54^.

#### Phylogenetic and network relationships among haplotypes

Maximum parsimony (MP), maximum-likelihood (ML) and Bayesian inference (BI) phylogenetic analyses were used to identify major clades and to evaluate the relationships between the haplotypes separately. MP analyses were performed in PAUP* 4.10b^55^ using a heuristic search with 1000 random sequence repetitions and tree-bisection-reconnection (TBR) branch swapping. Consensus trees (50% majority rule) were obtained if more than one equally parsimonious tree was found. ModelTest 3.7^56^ and the Akaike information criterion (AIC)^57^ were used to identify the appropriate nucleotide substitution models, and the selected models of sequence evolution were used for the ML phylogeny reconstruction. The ML analysis was performed in PAUP* 4.10b using a heuristic search strategy with 10 random additions of sequences and TBR branch swapping. Bootstrap analysis was performed under the same model with 100 pseudoreplicates, 10 random addition sequences per replicate and TBR branch swapping. Bayesian analysis was also carried out using the model selected by Modeltest 3.7. The analysis used a random starting tree and proceeded for one million Markov chain Monte Carlo generations, and trees were sampled every 100 generations. The first 2,500 generations (25% of the total) were later discarded as burn-in; 50% majority-rule consensus trees were generated from the remaining trees and posterior probabilities computed. Compared with conventional phylogenetic trees, haplotype networks can enhance the relationships among haplotypes and are preferable for intraspecific analyses. The median-joining network of the haplotypes of *COI* and *Cytb* were generated in Network 2.0 by a median-joining method^58^.

#### Genetic differentiation and population genetic structure

Analysis of molecular variance (AMOVA) and calculation of population genetic differentiation (*F*_ST_) statistics between the populations were conducted in Arlequin 3.0^59^. Mantel tests were performed to determine the significance between genetic and geographic distances.

#### Neutrality test and demographic history

We performed codon-based *Z*-tests of selection to estimate the probabilities of neutral (dN = dS), positive (dN > dS) and purifying selection (dN < dS) using MEGA 6^53^. Demographic history changes were analyzed for *A. gossypii* using two neutrality tests, Tajima’s *D* ^29^ and Fu’s *F*_S_^30^, which were calculated from the total number of segregating sites and used to assess evidence for population expansion. Mismatch distributions were calculated between the observed and expected mismatch distributions used as a test statistic. According to coalescent theory, a population usually exhibits a unimodal mismatch distribution following a recent population demographic or range expansion^60^. To determine whether movement patterns were influenced by spatial scale, we conducted a spatial analysis of genetic structure by performing the isolation by distance (IBD) analysis for each site type using a Mantel test implemented in the web-based IBDWS 3.32^61^. An IBD analysis was used to determine the relationship between calculated pairwise *F*_ST_ and pairwise geographic distances (km) between collection sites.

### Microsatellite data

#### Gene variation and genetic diversity

Genotypic linkage disequilibrium (LD) was tested among all pairs of loci across all populations using GenePop 3.4^62^ and exact probability tests. An exact test for HWE was conducted per locus and over all loci in each population using the same program. Corrections for multiple tests were performed by Bonferroni corrections. Null allele frequencies for each microsatellite locus were estimated following the expectation maximum algorithm of Dempster *et al*.^63^ using FreeNA^41^. The excluding null alleles (ENA) correction method was used to provide accurate estimation of *F*_ST_ in presence of null alleles. The population genetic diversity indices such as mean number of alleles (*N*_a_), effective number of alleles (*N*_e_), Shannon’s information index (*I*); observed heterozygosity (*H*_o_), expected heterozygosity (*H*_e_), and unbiased expected heterozygosity (uH_E_) were assessed using GenAlEx 6.41^64^. Allelic richness (*A*_R_), fixation index (*F*_ST_), inbreeding coefficient (*F*_IS_) among these regions were calculated in FSTAT 2.9.3^65^. The levels of genetic differentiation between pairs of populations were also estimated in Arlequin 3.0^59^.

#### Population genetic structure

Population structure was estimated using principal coordinate analysis (PCoA) implemented in GenAlex 6.41^64^ and by using the Bayesian model-based clustering method implemented in STRUCTURE 2.2.3^66^. We also used POPTREE 2^67^ to construct a unrooted tree using the neighbour-joining method. PCoA was based on the covariance of the genetic distance matrix. STRUCTURE sorts individuals into *K* optimal region groups, according to their genetic similarity. The allele frequencies of different populations were assumed to be correlated due to historical migration and common ancestry^68^. In addition, the admixture model of individual ancestry was used to assign hybrid individuals into population clusters; no prior population information was used^69^. Ten independent runs for *K* values ranging from 1 to 10 were performed with a burn-in length of 30,000 followed by 1,000,000 Markov chain Monte Carlo replicates. To identify the optimal number of groups (*K*), measures of change in the likelihood function (*ΔK*) and *F*_ST_ (*ΔF*_ST_) were calculated using the package CORRSIEVE6^70, 71^, and the plateau criterion was applied^57^. The software CLUMPP 1.1^72^ was used for model averaging of individual ancestry coefficients across the 10 independent runs. Then clusters were visualized using DISTRUCT 1.1^73^. The consistency of the clusters identified through the STRUCTURE approach was tested by a dissimilarity analysis, which was calculated from allelic data. The significance of the hierarchical partitioning of genetic structure among the geographic groups was examined using an analysis of molecular variance (AMOVA) in Arlequin 3.0^59^. Three hierarchical levels were defined: (1) between regional groups (western and eastern region), (2) between populations within regional groups, (3) between individuals within populations. BayesAss 3^74^ was used to estimate recent migration between regional groups. We ran the MCMC for 20,000,000 iterations, discarding the first 2,000,000 iterations and sampling per 200 iterations. We then adjusted the mixing parameters to ensure an acceptance rate ofabout 30% and carried out three independent runs with different random number seeds. Additionally, we conducted an IBD analysis between microsatellite and geographic distance data using a Mantel test implemented in IBDWS 3.23^61^.

#### Bottleneck analysis

We tested the bottleneck effect at the population level to explore population demography using different models and testing methods implemented in Bottleneck 1.2.02, and the infinite allele model (IAM), two-phase model (TPM), and the strict stepwise mutation model (SMM) were applied with 10,000 replications^75^. A qualitative descriptor of the allele frequency distribution (mode-shift indicator), which discriminates between bottlenecked and stable populations was also used. Under the three models, the standardized differences test was removed from this study because this test is typically only used when at least 20 polymorphic loci are available.

## Electronic supplementary material

Revised supplementary information files

## References

[1] Schirmer S, Sengonca C, Blaeser P (2008). Influence of abiotic factors on some biological and ecological characteristics of the aphid parasitoid *Aphelinus asychis* (Hymenoptera: Aphelinidae) parasitizing *Aphis gossypii* (Sternorrhyncha: Aphididae). Eur. J. Entomol..

[2] Kersting U, Satar S, Uygun N (1999). Effect of temperature on development rate and fecundity of apterous *Aphis gossypii* Glover (Hom., Aphididae) reared on *Gossypium hirsutum* L. J. Appl. Entomol.

[3] Ebert TA, Cartwright B (1997). Biology and ecology of *Aphis gossypii* Glover (Homoptera: aphididae). Southwest. Entomol..

[4] Inaizumi M (1981). Life cycle of *Aphis gossypii* (Homoptera, Aphididae) with special reference to biotype differentiation on various host plants. Jpn. J. Entomol..

[5] Guldemond JA, Tigges WT, De Vrijer WF (1994). Host races of *Aphis gossypii* (Homoptera, Aphididae) on cucumber and chrysanthemum. Environ. Entomo..

[6] Wool D, Hales D, Sunnucks P (1995). Host plant relationships of *Aphis gossypii* Glover (Homoptera: aphididae) in Australia. J. Aust. Entomol. Soc..

[7] Hales DF, Tomiuk J, Wöhrmann K, Sunnucks P (1997). Evolutionary and genetic aspects of aphid biology: A review. Eur. J. Entomol..

[8] Blackman RL (1994). The simplification of aphid terminology. Eur. J. Entomol..

[9] Delorme R, Augé D, Béthenod MT, Villatte F (1997). Communication to the editor insecticide resistance in a strain of *Aphis gossypii* from Southern France. Pestic. Sci..

[10] Herron GA, Powis K, Rophail J (2001). Insecticide resistance in *Aphis gossypii* Glover (Hemiptera: Aphididae), a serious threat to Australian cotton. Aust. J. Entomol..

[11] Ahmad M, Arif MI, Denholm I (2003). High resistance of field populations of the cotton aphid *Aphis gossypii* Glover (Homoptera: phididae) to pyrethroid insecticides in Pakistan. J. Econ. Entomol..

[12] Ai Y, Qiu XH, He FQ (2003). A review of insecticide resistance mechanism of *Aphis gossypii*. Entomol Knowl.

[13] Sun JT, Lian C, Navajas M, Hong XY (2012). Microsatellites reveal a strong subdivision of genetic structure in Chinese populations of the mite *Tetranychus urticae* Koch (Acari: Tetranychidae). BMC Genet..

[14] Susanta KB (2006). Molecular marker systems in insects: current trends and future avenues. Mol. Ecol..

[15] Simon JC (1999). Reproductive mode and population genetic structure of the cereal aphid *Sitobion avenae* studied using phenotypic and microsatellite markers. Mol. Ecol..

[16] Massonnet B, Leterme N, Simon JC, Weisser WW (2001). Characterization of microsatellite loci in the aphid species *Macrosiphoniella tanacetaria* (Homoptera, Aphididae). Mol. Ecol. Notes..

[17] Llewellyn KS (2003). Migration and genetic structure of the grain aphid (*Sitobion avenae*) in Britain related to climate and clonal fluctuation as revealed using microsatellites. Mol. Ecol..

[18] Astorga M, Galleguillos R (1998). Genetic divergence of jack mackerel of genus *Trachurus* from northwestern and southeastern Pacific. Rev. Biol. Mar. Oceanog..

[19] Barraclough TG, Nee S (2001). Phylogenetic and speciation. Trends. Ecol. Evol..

[20] Zhang F, Liu XD (2012). Variation of host-specialized and migratory biotypes of *Aphis gossypii* Glover based on mtDNA COI gene sequences. J. Nanjing Agr. Univ..

[21] Vanlerberghe-Masutti F, Chavigny P (1998). Host-based genetic differentiation in the aphid *Aphis gossypii* Glover, evidenced from RAPD fingerprints. Mol. Ecol..

[22] Fuller SJ, Chavigny P, Lapchin L, Vanlerberghe-Masutti F (1999). Variation in clonal diversity in glasshouse infestations of the aphid, *Aphis gossypii* Glover in southern France. Mol. Ecol..

[23] Charaabi K (2008). Genotypic diversity of the cotton-melon aphid *Aphis gossypii* (Glover) in Tunisia is structured by host plants. B. Entomol. Res..

[24] Takada H (1988). Interclonal variation in photoperiodic response for sexual morph production of Japanese *Aphis gossypii* Glover (Hemiptera, Aphididae). J. Appl. Entomol..

[25] Gong P, Yang XW, Zhang XX, Liu XD, Chen XF (2001). Microsatellite primer-PCR studies on the population differentiation of *Aphis gossypii* in relation to host plants and seasons. Acta. Ecol. Sin..

[26] Gong P, Zhang XX, Yang XW, Chen XF (2001). Microsatellite DNA polymorphism in different forms of the cotton aphid. Acta. Ecol. Sin..

[27] Brévault T, Carletto J, Linderme D, Vanlerberghe-Masutti F (2008). Genetic diversity of the cotton aphid *Aphis gossypii* in the unstable environment of a cotton growing area. Agr. Forest. Entomol..

[28] Thomas S (2008). Genetic diversity of the cotton-melon aphid, *Aphis gossypii* Glover in different melon growing areas of France. Redia..

[29] Tajima F (1989). Statistical method for testing the neutral mutation hypothesis by DNA Polymorphism. Genetics.

[30] Fu YX (1997). Statistical tests of neutrality of mutations against population growth, hitchhiking and background selection. Genetics.

[31] Delmotte F, Leterme N, Gauthier JP, Rispe C, Simon JC (2002). Genetic architecture of sexual and asexual populations of the aphid *Rhopalosiphum padi* based on allozyme and microsatellite markers. Mol. Ecol..

[32] Sunnucks P, England PR, Taylor AC, Hales DF (1996). Microsatellite and chromosome evolution of parthenogenetic *Sitobion* aphids in Australia. Genetics.

[33] Normark BB (1999). Evolution in a putatively ancient asexual aphid lineage: recombination and rapid karyotype change. Evol..

[34] Wilson AC, Sunnucks P, Hales DF (1999). Microevolution, low clonal diversity and genetic affinities of parthenogenetic *Sitobion aphids* in New Zealand. Mol. Ecol..

[35] Vanlerberghe-Masutti F, Chavigny P, Fuller SJ (1999). Characterization of microsatellite loci in the aphid species *Aphis gossypii* Glover. Mol. Ecol..

[36] Timm AE, Pringle KL, Warnich L (2005). Genetic diversity of woolly apple aphid *Eriosoma lanigerum* (Hemiptera: Aphididae) populations in the Western Cape. South Africa. B. Entomol. Res..

[37] Zamoum T (2005). Does insecticide resistance alone account for the low genetic variability of asexually reproducing populations of the peach-potato aphid *Myzus persicae*?. Heredity..

[38] Posada D, Crandall KA (2001). Intraspecific gene genealogies: trees grafting into networks. Trends. Ecol. Evol..

[39] Gascon C (2000). Riverine barriers and the geographic distribution of Amazonian species. P. Natl. Acad. Sci. USA..

[40] Cozzolino S, Cafasso D, Pellegrino G, Musacchio A, Widmer A (2003). Fine-scale phylogeographical analysis of *Mediterranean Anacamptis* palustris (Orchidaceae) populations based on chloroplast minisatellite and microsatellite variation. Mol. Ecol..

[41] Chapuis MP, Estoup A (2007). Microsatellite null alleles and estimation of population differentiation. Mol. Biol. Evol..

[42] Yuan JH, Cheng FY, Zhou SL (2012). Genetic structure of the tree peony (Paeonia rockii) and the Qinling Mountains as a geographic barrier driving the fragmentation of a large population. PLoS One.

[43] Peterson MA, Denno RF (1998). The influence of dispersal and diet breadth on patterns of genetic isolation by distance in phytophagous insects. Am. Nat..

[44] Meinke LJ, Ware GW (1978). Tolerance of three beet armyworm strains in Arizona to methomyl. J. Econ. Entomol..

[45] Chaufaux J, Ferron P (1986). Sensibilite differente de deux populations de *Spodoptera exigua* Hubner (Lepidoptera: Noctuidae) aux baculovirus et aux pyrethrinoides de synthese. Agronomie.

[46] Moores GD, Gao XW, Denholm I, Devonshire AL (1996). Characterization of insensitive acetylcholinesterase in insecticideresistant cotton aphids, *Aphis gossypii* Glover (Homoptera: Aphididae). Pestic. Biochem. Phys..

[47] Favret C, Voegtlin DJ (2004). Speciation by host-switching in *Cinara* (Insecta: Hemiptera: Aphididae). Mol. Phylogenet. Evol..

[48] Harry M, Solignac M, Lachaise D (1998). Molecular evidence for parallel evolution of adaptive syndromes in fig-breeding *Lissocephala* (Drosophilidae). Mol. Phylogenet. Evol..

[49] Michel AP, Zhang W, Jinkyo J, Kang ST, Mian MAR (2009). Population genetic structure of *Aphis glycines*. Environ. Entomol..

[50] Kim KS, Hill CB, Hartman GL, Mar M, Diers BW (2008). Discovery of soybean aphid biotypes. Crop. Sci..

[51] Gauffre B, D´Acier AC (2006). New polymorphic microsatellite loci, cross-species amplification and PCR multiplexing in the black aphid, *Aphis fabae* Scopoli. Mol. Ecol. Notes..

[52] Thompson JD, Gibson TJ, Plewniak F, Jeanmougin F, Higgins DG (1997). The CLUSTAL X windows interface: flexible strategies for multiple sequence alignment aided by quality analysis tools. Nucleic. Acids. Res..

[53] Tamura K, Stecher G, Peterson D, Filipski A, Kumar S (2013). MEGA6: Molecular evolutionary genetics analysis version 6.0. Mol. Biol. Evol..

[54] Rozas JS, Sánchez-DelBarrio JC, Messegyer X, Rozas R (2003). DnaSP: DNA polymorphism analyses by the coalescent and other methods. Bioinformatics..

[55] Swofford, D. L. *PAUP*. Phylogenetic analysis using parsimony* (**and other methods*)*, Version 4.0*. Sinauer Associates, Sunderland, Massachusetts (2003).

[56] Posada D, Crandall KA (1998). MODELTEST: testing the model of DNA substitution. Bioinformatics.

[57] Posada D, Buckley TR (2004). Model selection and model averaging in phylogenetics: advantages of Akaike information criterion and Bayesian approaches over likelihood ratio tests. Syst. Biol..

[58] Bandelt HJ, Forster AR, Röhl A (1999). Median-joining networks for inferring intraspecific phylogenies. Mol. Biol. Evol..

[59] Excoffier L, Laval G, Schneider S (2005). Arlequin, ver. 3.0: an integrated software package for population genetics data analysis. Evol Bioinform Online..

[60] Rogers AR, Harpending H (1992). Population growth makes waves in the distribution of pairwise genetic differences. Mol. Biol. Evol..

[61] Jensen JL, Bohonak AJ, Kelley ST (2005). Isolation by distance, web service. BMC Genet..

[62] Raymond M, Rousset F (1995). GENEPOP (version 1.2): Population genetics software for exact tests and ecumenicism. J. Hered..

[63] Dempster AP, Laird NM, Rubin DB (1977). Maximum likelihood from incomplete data via the EM-algorithm. J. R. Stat. Soc. B..

[64] Peakall R, Smouse PE (2006). GENALEX 6: genetic analysis in Excel. Population genetic software for teaching and research. Mol. Ecol. Notes..

[65] Goudet J (1995). FSTAT (version 1.2): a computer program to calculate F-statistics. J. Hered..

[66] Pritchard JK, Stephens M, Donnelly P (2000). Inference of population structure using multilocus genotypic data. Genetics.

[67] Takezaki N, Nei M, Tamura K (2010). POPTREE 2: Software for constructing population trees from allele frequency data and computing other population statistics with Windows-interface. Mol. Bio. Evol..

[68] Falush D, Stephens M, Pritchard JK (2003). Inference of population structure using multilocus genotype data: linked loci and correlated allele frequencies. Genetics.

[69] Pritchard, J. & Falush, D. Documentation for structure software: version 2.2. (Chicago, USA, University of Chicago, 2007).

[70] Evanno G, Regnaut S, Goudet J (2005). Detecting the number of clusters of individuals using the software STRUCTURE: a simulation study. Mol. Ecol..

[71] Campana MG, Hunt HV, Jones H, White J (2011). CorrSieve: software for summarizing and evaluating structure output. Molecular Ecology Resources.

[72] Jakobsson M, Rosenberg NA (2007). CLUMPP: a cluster matching and permutation program for dealing with label switching and multimodality in analysis of population structure. Bioinform..

[73] Rosenberg NA (2004). Distruct: a program for the graphical display of population structure. Mol. Ecol. Notes..

[74] Wilson GA, Rannala B (2003). Bayesian inference of recent migration rates using multilocus genotypes. Genetics.

[75] Piry S, Luikart G, Cornuet JM (2001). Bottleneck: A computer program for detecting recent reductions in the effective population size using allele frequency data. J. Hered..

