# Supplementary material for: Genetic variation and phylogeographic structure of the cotton aphid, Aphis gossypii, based on mitochondrial DNA and microsatellite markers
Source: Sci Rep. 2017 May 15;7:1920. doi: 10.1038/s41598-017-02105-4 (PMC5432507; doi:10.1038/s41598-017-02105-4)
Supplement: Supplementary file 1 — Revised supplementary information files [file 41598_2017_2105_MOESM1_ESM.doc]

**Genetic variation and phylogeographic structure of the cotton aphid, *Aphis gossypii*, based on mitochondrial DNA and microsatellite markers**

Xing-Ya Wang^1, 2+^, Xian-Ming Yang^1+^, Bin Lu^3+^, Li-Hong Zhou ^4^ & Kong-Ming Wu^1*^

^1^ State Key Laboratory for Biology of Plant Diseases and Insect Pests, Institute of Plant Protection, Chinese Academy of Agricultural Sciences, Beijing 100193, P.R. China

^2^ Plant Protection College, Shenyang Agricultural University, Shenyang, Liaoning, 110866, P.R. China

^3^ Chengdu Institute of Biology, Chinese Academy of Sciences, Chengdu, Sichuan, 610041, P.R. China

^4^ Institute of Flower Research, Liaoning Academy of Agricultural Sciences, Shenyang, Liaoning 110161, P.R. China

^+^ These authors contributed equally to this work.

**^*^ Correspondence authors:**

Kongming Wu

Email: [kmwu@ippcaas.cn](mailto:kmwu@ippcaas.cn)

Tel: +86(0)1082105551

Mailing address: State Key Laboratory for Biology of Plant Diseases and Insect Pests. Institute of Plant Protection, Chinese Academy of Agricultural Sciences, Beijing 100193, P.R. China.

**Supplementary Data**

**Figure Legends**

**Supplementary Fig. S1** Phylogenetic trees of *Aphis gossypii* based on haplotype sequence variation of the combined *COI* and *Cytb* sequences. Bayesian tree (a); maximum-likelihood (ML) tree (b); bootstrap percentages from ML analysis and posterior probabilities of Bayesian analysis are shown above the branch (ML and Bayesian analyses: best-fit model HKY+G). *Aphis glycines* (GenBank accession nos. JQ860254 and GU205350) and *Aphis craccivora* (GenBank accession nos. AB506714 and AM085376) were used as outgroup species.

**Supplementary Fig. S2** Bayesian inference for the other estimated cluster proportion (*K* = 3 and *K* = 4) using STRUCTURE for *Aphis gossypii*. Error bars represent standard deviations over 10 runs. For the assignment proportion in all populations, each individual is represented by a thin vertical line, which was partitioned into *K* segments that represent its estimated population group membership fractions. Population codes are given in Table 2.

**Supplementary Fig. S3** Analyses of mismatch distributions and the neutrality test results of Tajima’s *D* and Fu’s *F*_S_ tests for sampling localities in China based on the combined *COI* and *Cytb* sequences (a, total population; b, eastern region; c, western region) The *x*-axis represents the numbers of pairwise differences; the *y*-axis represents the relative frequency. The expected distribution under a model of population expansion is given as a continuous line, and the observed distribution is given as a dashed line.

**Supplementary Fig. S1**


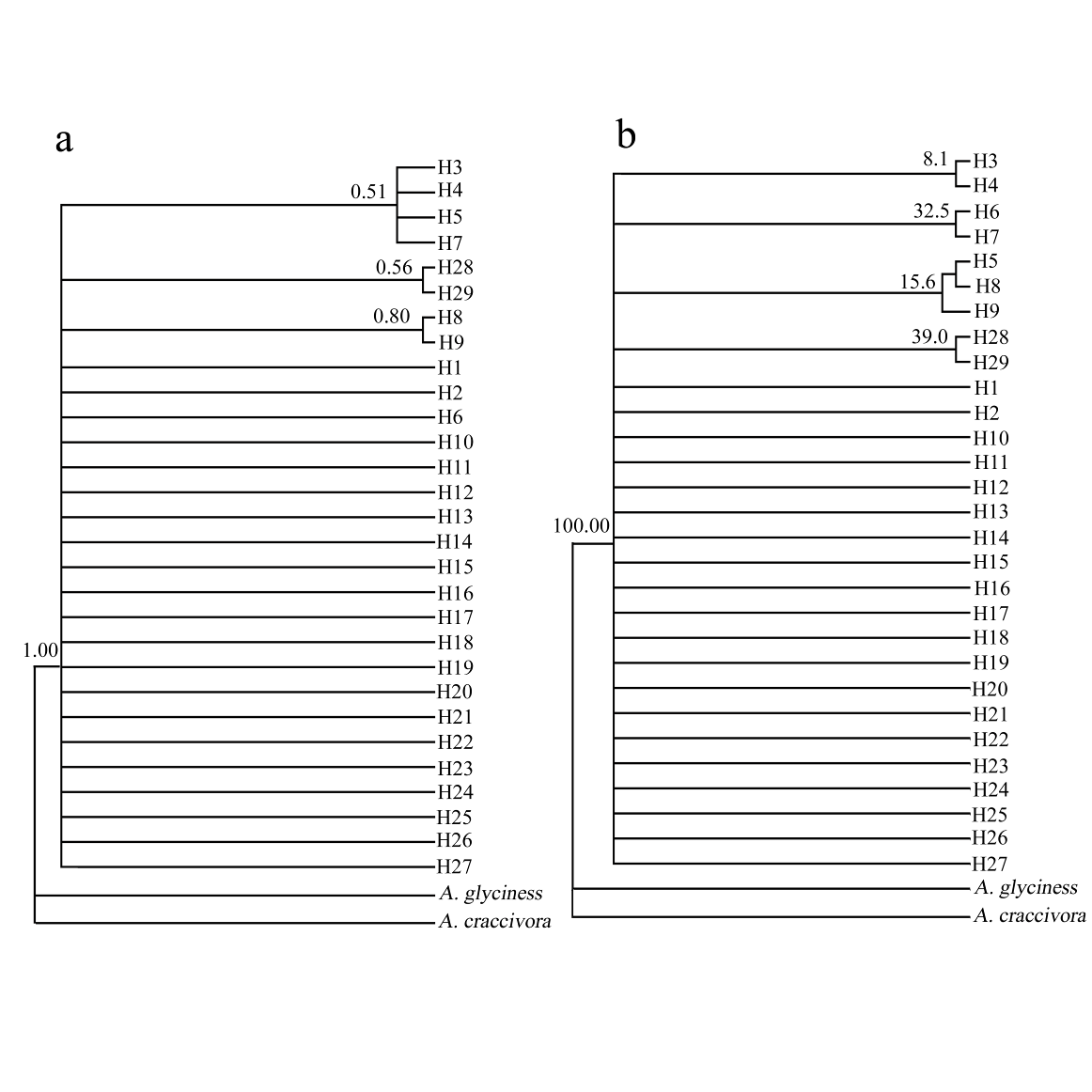


**Supplementary Fig. S2**


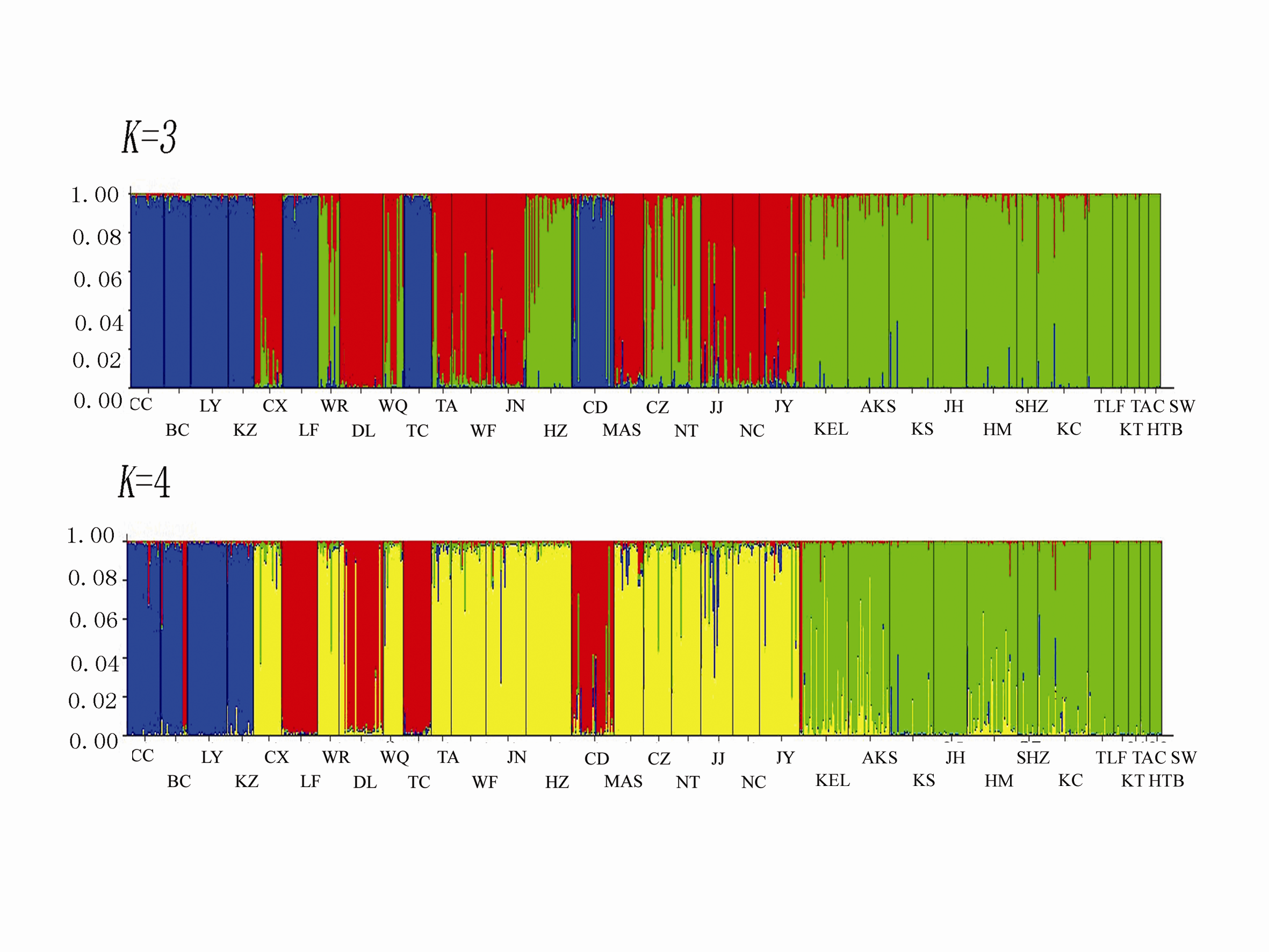


**Supplementary Fig. S3**

**
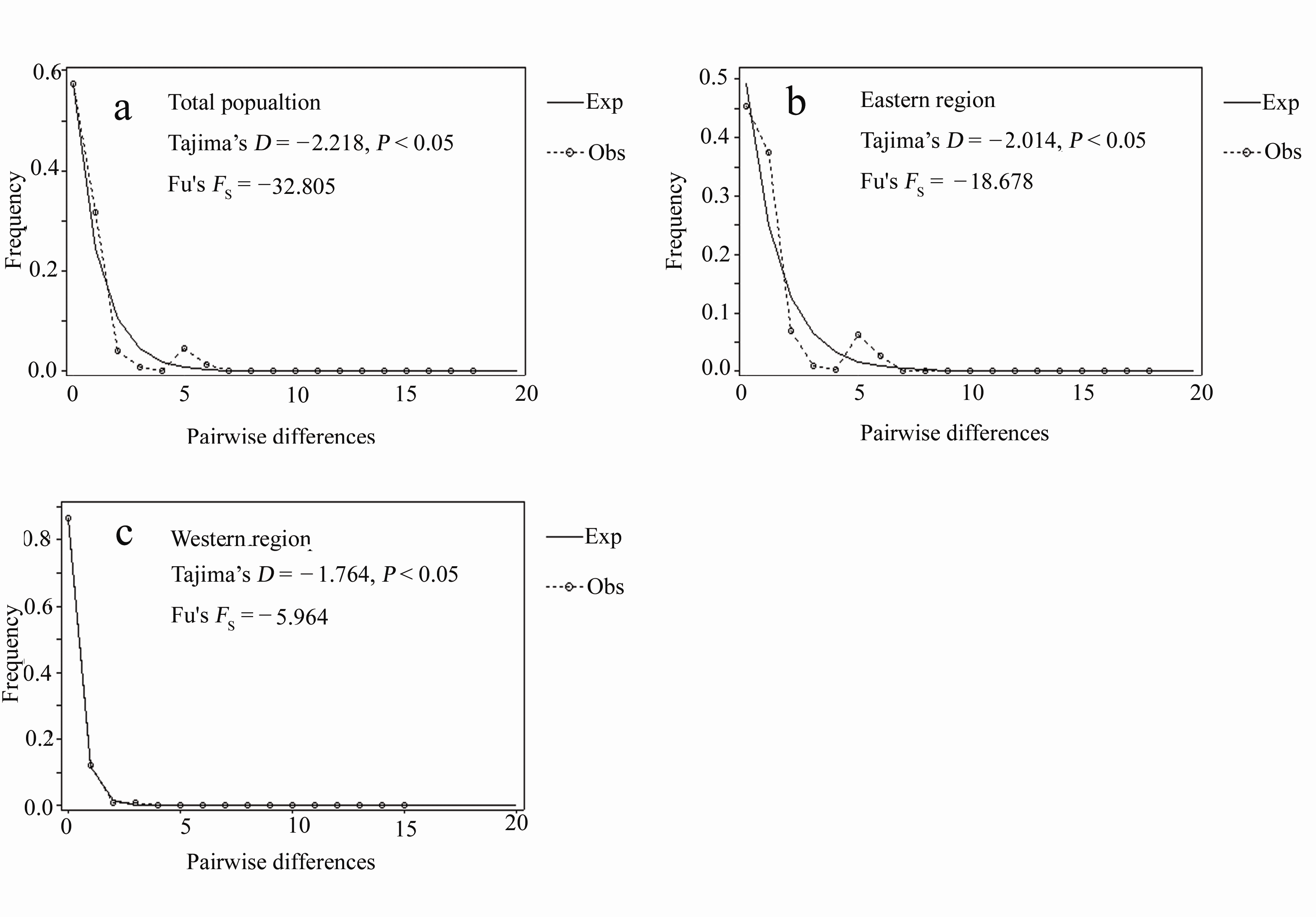
**

**Supplementary Table S1** Estimates of null allele frequency for each locus

| Locus | Null allele frequency |
| --- | --- |
| 1 | 0.046 |
| 2 | 0.050 |
| 3 | 0.077 |
| 4 | 0.039 |
| 5 | 0.079 |
| 6 | 0.167 |
| 7 | 0.056 |
| 8 | 0.071 |
| 9 | 0.084 |
| 10 | 0.027 |

**Supplementary Table S2** Estimates of *F*_ST_ without and with the ENA correction for each locus

| Locus | *F*_ST_ not using ENA | *F*_ST_ using ENA |
| --- | --- | --- |
| 1 | 0.131 | 0.129 |
| 2 | 0.525 | 0.512 |
| 3 | 0.150 | 0.149 |
| 4 | 0.193 | 0.183 |
| 5 | 0.184 | 0.176 |
| 6 | 0.193 | 0.162 |
| 7 | 0.474 | 0.441 |
| 8 | 0.088 | 0.082 |
| 9 | 0.136 | 0.131 |
| 10 | 0.170 | 0.162 |
| Average | 0.224 | 0.213 |
| All loci | 0.198 | 0.190 |

The excluding null alleles (ENA).

**Supplementary Table S3 List of populations of *Aphis gossypii* studied indicating the genetic diversity at 10 microsatellite loci**

| Pop | *N*_a_ | *N*_e_ | *I* | *H*_o_ | *H*_e_ | uH_E_ | *F* | *F*_IS_ | *A*_R_ |
| --- | --- | --- | --- | --- | --- | --- | --- | --- | --- |
| CC | 8.400 | 3.765 | 1.422 | 0.524 | 0.614 | 0.626 | 0.194 | 0.166 | 4.194 |
| BC | 8.400 | 4.076 | 1.537 | 0.469 | 0.661 | 0.680 | 0.290 | 0.317 | 4.615 |
| LY | 5.300 | 2.184 | 0.905 | 0.291 | 0.433 | 0.441 | 0.428 | 0.345 | 2.912 |
| KZ | 6.500 | 3.449 | 1.280 | 0.472 | 0.578 | 0.593 | 0.169 | 0.210 | 3.916 |
| CX | 7.200 | 3.277 | 1.386 | 0.652 | 0.651 | 0.663 | 0.031 | 0.017 | 3.969 |
| LF | 4.500 | 2.592 | 0.965 | 0.532 | 0.476 | 0.496 | –0.113 | –0.075 | 3.192 |
| WR | 8.000 | 3.449 | 1.473 | 0.527 | 0.667 | 0.680 | 0.231 | 0.234 | 4.244 |
| DL | 6.600 | 3.840 | 1.393 | 0.539 | 0.647 | 0.662 | 0.285 | 0.189 | 4.112 |
| WQ | 7.600 | 3.689 | 1.440 | 0.539 | 0.642 | 0.658 | 0.235 | 0.185 | 4.276 |
| TC | 6.700 | 3.549 | 1.438 | 0.658 | 0.681 | 0.698 | 0.055 | 0.059 | 4.176 |
| TA | 6.700 | 4.129 | 1.455 | 0.537 | 0.653 | 0.679 | 0.178 | 0.216 | 4.567 |
| WF | 6.800 | 3.318 | 1.352 | 0.518 | 0.637 | 0.650 | 0.186 | 0.207 | 3.909 |
| JN | 8.000 | 3.719 | 1.466 | 0.697 | 0.668 | 0.680 | 0.005 | –0.026 | 4.184 |
| HZ | 4.500 | 2.013 | 0.748 | 0.506 | 0.388 | 0.395 | –0.252 | –0.286 | 2.455 |
| CD | 7.500 | 3.796 | 1.427 | 0.555 | 0.635 | 0.651 | 0.105 | 0.159 | 4.404 |
| MAS | 4.800 | 2.896 | 1.011 | 0.522 | 0.485 | 0.499 | –0.091 | –0.048 | 3.260 |
| CZ | 5.800 | 2.522 | 1.014 | 0.504 | 0.472 | 0.483 | –0.032 | –0.045 | 3.212 |
| NT | 7.400 | 3.774 | 1.397 | 0.563 | 0.616 | 0.629 | 0.164 | 0.108 | 4.173 |
| JJ | 5.300 | 2.677 | 1.065 | 0.427 | 0.524 | 0.541 | 0.234 | 0.215 | 3.319 |
| NC | 6.600 | 3.519 | 1.287 | 0.507 | 0.599 | 0.610 | 0.180 | 0.171 | 3.828 |
| JY | 5.600 | 2.845 | 1.051 | 0.635 | 0.511 | 0.519 | –0.231 | –0.287 | 3.020 |
| KEL | 6.500 | 3.398 | 1.279 | 0.489 | 0.587 | 0.599 | 0.168 | 0.181 | 3.839 |
| AKS | 5.900 | 3.171 | 1.184 | 0.501 | 0.557 | 0.568 | 0.199 | 0.121 | 3.579 |
| KS | 5.700 | 2.757 | 1.032 | 0.500 | 0.495 | 0.503 | 0.184 | –0.005 | 3.119 |
| JH | 5.800 | 3.352 | 1.203 | 0.521 | 0.568 | 0.580 | 0.159 | 0.115 | 3.627 |
| HM | 5.600 | 2.732 | 1.090 | 0.596 | 0.530 | 0.538 | –0.104 | –0.109 | 3.272 |
| SHZ | 5.700 | 3.225 | 1.252 | 0.473 | 0.588 | 0.609 | 0.226 | 0.206 | 3.344 |
| KC | 7.200 | 3.335 | 1.259 | 0.439 | 0.559 | 0.566 | 0.193 | 0.226 | 3.754 |
| TLF | 5.200 | 2.961 | 1.153 | 0.572 | 0.569 | 0.584 | 0.072 | –0.037 | 3.427 |
| KT | 4.400 | 3.099 | 1.123 | 0.510 | 0.563 | 0.590 | 0.093 | 0.142 | 3.606 |
| TAC | 3.900 | 2.943 | 1.070 | 0.502 | 0.562 | 0.593 | 0.102 | 0.208 | 3.241 |
| HTB | 3.900 | 3.202 | 1.112 | 0.414 | 0.577 | 0.625 | 0.292 | 0.359 | 3.696 |
| SW | 4.100 | 3.130 | 1.084 | 0.488 | 0.550 | 0.585 | 0.145 | 0.174 | 3.601 |
| Eastern region group | 20.600 | 5.203 | 1.938 | 0.507 | 0.708 | 0.745 | 0.317 | 0.276 | 20.996 |
| Western region group | 18.900 | 4.313 | 1.663 | 0.532 | 0.628 | 0.664 | 0.276 | 0.204 | 12.939 |
| Total popualtion | 26.100 | 5.027 | 1.900 | 0.524 | 0.719 | 0.720 | 0.298 | 0.273 | 21.410 |

Abbreviations: *N*_a_, observed number of alleles; *N*_e_*,* effective number of alleles; *I*, Shannon’s information index, *H*_o_, observed heterozygosity, *H*_e_, expected heterozygosity; uH_E_, unbiased expected heterozygosity; *F*, fixation index; *F*_IS_, inbreeding index; *A*_R_, allelic richness.

**Supplementary Table S4 Genetic diversity and neutrality test among different populations of *Aphis gossypii* based on the combined *COI* and *Cytb* sequences**

| Locality | *N* / *H* | Hd | Pi | Tajima’s *D* | Fu’s *F*_S_ |
| --- | --- | --- | --- | --- | --- |
| CC | 25 / 3 | 0.157 | 0.00013 | -1.514 | -2.128 |
| BC | 20 / 1 | 0.000 | 0.00000 | 0.000 | 0.000 |
| LY | 29 / 1 | 0.000 | 0.00000 | 0.000 | 0.000 |
| KZ | 16 / 4 | 0.575 | 0.00054 | -0.815 | -1.208 |
| CX | 27 / 1 | 0.000 | 0.00000 | 0.000 | 0.000 |
| LF | 12 / 2 | 0.530 | 0.00044 | 1.381 | 1.152 |
| WR | 32 / 4 | 0.333 | 0.00029 | -1.223 | -1.973 |
| DL | 21 / 3 | 0.343 | 0.00030 | -0.804 | -0.775 |
| WQ | 21 / 3 | 0.495 | 0.00043 | -0.133 | -0.090 |
| TC | 19 / 4 | 0.509 | 0.00064 | -1.432 | -0.648 |
| TA | 15 / 4 | 0.667 | 0.00066 | -0.395 | -0.825 |
| WF | 23 / 5 | 0.545 | 0.00051 | -1.194 | -2.219 |
| JN | 28 / 4 | 0.381 | 0.00034 | -1.115 | -1.704 |
| HZ | 32 / 4 | 0.526 | 0.00047 | -0.547 | -0.866 |
| CD | 20 / 5 | 0.568 | 0.00059 | -0.410 | -1.990 |
| MAS | 21 / 4 | 0.271 | 0.00024 | -1.727 | -2.820 |
| CZ | 21 / 4 | 0.633 | 0.00064 | -0.197 | -0.549 |
| NT | 24 / 3 | 0.453 | 0.00053 | -1.098 | 0.408 |
| JJ | 15 / 5 | 0.638 | 0.00074 | -1.409 | -1.808 |
| NC | 29 / 5 | 0.510 | 0.00053 | -1.357 | -1.828 |
| JY | 33 / 2 | 0.436 | 0.00180 | 2.045^*^ | 6.789 |
| KEL | 32 / 3 | 0.123 | 0.00010 | -1.504 | -2.437 |
| AKS | 29 / 1 | 0.000 | 0.00000 | 0.000 | 0.000 |
| KS | 30 / 4 | 0.193 | 0.00033 | -1.873^*^ | -1.747 |
| JH | 22 /2 | 0.247 | 0.00020 | -0.175 | 0.303 |
| HM | 35 / 2 | 0.111 | 0.00009 | -0.807 | -0.572 |
| SHZ | 12 / 3 | 0.318 | 0.00028 | -1.451 | -1.325 |
| KC | 38 / 1 | 0.000 | 0.00000 | 0.000 | 0.000 |
| TLF | 22 / 2 | 0.455 | 0.00038 | 1.142 | 1.274 |
| KT | 11 / 1 | 0.000 | 0.00000 | 0.000 | 0.000 |
| TAC | 9 / 1 | 0.000 | 0.00000 | 0.000 | 0.000 |
| HTB | 6 / 1 | 0.000 | 0.00000 | 0.000 | 0.000 |
| SW | 9 / 2 | 0.222 | 0.00018 | -1.088 | -0.263 |
| eastern region group | 483 / 25 | 0.549 | 0.00086 | -2.014^**^ | -18.678 |
| western region group | 255 / 7 | 0.141 | 0.00014 | -1.764^**^ | -5.964 |
| Total population | 738 / 29 | 0.420 | 0.00062 | -2.218^**^ | -32.805 |

*N*, sample size; *H*, number of haplotypes; Hd, haplotype diversity; Pi, nucleotide diversity; ^*^*P* < 0.05; ^**^*P* < 0.01.

***Supplementary Table S5*** *Population pairwise F_ST_ values based on 10 microsatellite loci between the populations of Aphis gosypii*

| Pop | CC | BC | LY | KZ | CX | LF | WR | DL | WQ | TC | TA | WF | JN | HZ | CD | MAS | CZ | NT | JJ | NC | JY | KEL | AKS | KS | JH | HM | SHZ | KC | TLF | KT | TAC | HTB |
| --- | --- | --- | --- | --- | --- | --- | --- | --- | --- | --- | --- | --- | --- | --- | --- | --- | --- | --- | --- | --- | --- | --- | --- | --- | --- | --- | --- | --- | --- | --- | --- | --- |
| CC |  |  |  |  |  |  |  |  |  |  |  |  |  |  |  |  |  |  |  |  |  |  |  |  |  |  |  |  |  |  |  |  |
| BC | 0.01* |  |  |  |  |  |  |  |  |  |  |  |  |  |  |  |  |  |  |  |  |  |  |  |  |  |  |  |  |  |  |  |
| LY | 0.09* | 0.09* |  |  |  |  |  |  |  |  |  |  |  |  |  |  |  |  |  |  |  |  |  |  |  |  |  |  |  |  |  |  |
| KZ | 0.03* | 0.06* | 0.11* |  |  |  |  |  |  |  |  |  |  |  |  |  |  |  |  |  |  |  |  |  |  |  |  |  |  |  |  |  |
| CX | 0.14* | 0.08* | 0.23* | 0.19* |  |  |  |  |  |  |  |  |  |  |  |  |  |  |  |  |  |  |  |  |  |  |  |  |  |  |  |  |
| LF | 0.32* | 0.25* | 0.37* | 0.30* | 0.23* |  |  |  |  |  |  |  |  |  |  |  |  |  |  |  |  |  |  |  |  |  |  |  |  |  |  |  |
| WR | 0.33* | 0.25* | 0.37* | 0.32* | 0.21* | 0.02* |  |  |  |  |  |  |  |  |  |  |  |  |  |  |  |  |  |  |  |  |  |  |  |  |  |  |
| DL | 0.16* | 0.12* | 0.19* | 0.22* | 0.14* | 0.38* | 0.31* |  |  |  |  |  |  |  |  |  |  |  |  |  |  |  |  |  |  |  |  |  |  |  |  |  |
| WQ | 0.14* | 0.07* | 0.18* | 0.15* | 0.08* | 0.11* | 0.12* | 0.07* |  |  |  |  |  |  |  |  |  |  |  |  |  |  |  |  |  |  |  |  |  |  |  |  |
| TC | 0.21* | 0.14* | 0.28* | 0.22* | 0.07 * | 0.06* | 0.06* | 0.20* | 0.06* |  |  |  |  |  |  |  |  |  |  |  |  |  |  |  |  |  |  |  |  |  |  |  |
| TA | 0.17* | 0.10* | 0.22* | 0.16* | 0.14* | 0.11* | 0.17* | 0.20* | 0.09* | 0.11* |  |  |  |  |  |  |  |  |  |  |  |  |  |  |  |  |  |  |  |  |  |  |
| WF | 0.15* | 0.11* | 0.23* | 0.16* | 0.07* | 0.12* | 0.16* | 0.19* | 0.08* | 0.06* | 0.05* |  |  |  |  |  |  |  |  |  |  |  |  |  |  |  |  |  |  |  |  |  |
| JN | 0.25* | 0.19* | 0.30* | 0.21* | 0.21* | 0.03* | 0.11 | 0.28* | 0.13* | 0.12 | 0.08* | 0.10* |  |  |  |  |  |  |  |  |  |  |  |  |  |  |  |  |  |  |  |  |
| HZ | 0.48* | 0.42* | 0.51* | 0.46* | 0.38* | 0.10* | 0.10 | 0.49* | 0.29* | 0.22* | 0.32* | 0.31* | 0.22* |  |  |  |  |  |  |  |  |  |  |  |  |  |  |  |  |  |  |  |
| CD | 0.20* | 0.12* | 0.26* | 0.19* | 0.11* | -0.01 | 0.05* | 0.21* | 0.06* | 0.04* | 0.02* | 0.06* | 0.06* | 0.15* |  |  |  |  |  |  |  |  |  |  |  |  |  |  |  |  |  |  |
| MAS | 0.32* | 0.24* | 0.38* | 0.29* | 0.22* | 0.01 | 0.02* | 0.38* | 0.13* | 0.07* | 0.09* | 0.13* | 0.06* | 0.12* | -0.02 |  |  |  |  |  |  |  |  |  |  |  |  |  |  |  |  |  |
| CZ | 0.39* | 0.32* | 0.45* | 0.38* | 0.29* | -0.01 | 0.07* | 0.44* | 0.23* | 0.15* | 0.22* | 0.22* | 0.14* | 0.13* | 0.10* | -0.01 |  |  |  |  |  |  |  |  |  |  |  |  |  |  |  |  |
| NT | 0.18* | 0.11* | 0.26* | 0.18* | 0.15* | 0.18 | 0.23* | 0.24* | 0.12* | 0.14* | 0.04* | 0.12* | 0.15* | 0.37* | 0.07* | 0.12* | 0.25* |  |  |  |  |  |  |  |  |  |  |  |  |  |  |  |
| JJ | 0.23* | 0.15* | 0.26* | 0.25* | 0.12* | 0.21* | 0.12* | 0.11* | 0.02 | 0.07* | 0.18* | 0.15* | 0.20* | 0.32* | 0.10* | 0.19* | 0.28* | 0.18* |  |  |  |  |  |  |  |  |  |  |  |  |  |  |
| NC | 0.20* | 0.11* | 0.24* | 0.22* | 0.07* | 0.20* | 0.15* | 0.11* | 0.04* | 0.05* | 0.13* | 0.13* | 0.21* | 0.33* | 0.09* | 0.18* | 0.27* | 0.12* | 0.02 |  |  |  |  |  |  |  |  |  |  |  |  |  |
| JY | 0.20* | 0.16* | 0.23* | 0.27* | 0.19* | 0.46* | 0.40* | 0.11* | 0.14* | 0.28* | 0.30* | 0.26* | 0.36* | 0.55* | 0.29* | 0.44* | 0.50* | 0.30* | 0.20* | 0.18* |  |  |  |  |  |  |  |  |  |  |  |  |
| KEL | 0.39* | 0.32* | 0.41* | 0.36* | 0.32* | 0.01 | 0.07* | 0.39* | 0.19* | 0.18* | 0.22* | 0.22* | 0.12* | 0.11* | 0.11* | 0.05* | 0.08* | 0.28* | 0.23* | 0.27* | 0.46* |  |  |  |  |  |  |  |  |  |  |  |
| AKS | 0.37* | 0.30* | 0.42* | 0.35* | 0.28* | -0.04 | 0.07* | 0.41* | 0.20* | 0.14* | 0.20* | 0.19* | 0.11* | 0.11* | 0.09* | 0.00 | 0.03* | 0.25* | 0.25* | 0.26* | 0.47* | 0.01 |  |  |  |  |  |  |  |  |  |  |
| KS | 0.40* | 0.33* | 0.43* | 0.37* | 0.37* | 0.14* | 0.22* | 0.44* | 0.24* | 0.27* | 0.22* | 0.26* | 0.18* | 0.29* | 0.16* | 0.13* | 0.17* | 0.26* | 0.32* | 0.32* | 0.50* | 0.08* | 0.10* |  |  |  |  |  |  |  |  |  |
| JH | 0.43* | 0.35* | 0.46* | 0.40* | 0.37* | 0.12* | 0.18* | 0.46* | 0.26* | 0.26* | 0.23* | 0.29* | 0.19* | 0.25* | 0.15* | 0.08* | 0.12* | 0.25* | 0.32* | 0.32* | 0.53* | 0.08* | 0.08* | 0.03* |  |  |  |  |  |  |  |  |
| HM | 0.42* | 0.35* | 0.45* | 0.40* | 0.34* | 0.04* | 0.09* | 0.42* | 0.23* | 0.20* | 0.23* | 0.25* | 0.13* | 0.15* | 0.11* | 0.04* | 0.05* | 0.28* | 0.28* | 0.29* | 0.51* | 0.01* | 0.02* | 0.10* | 0.05* |  |  |  |  |  |  |  |
| SHZ | 0.46* | 0.38* | 0.48* | 0.43* | 0.43* | 0.31* | 0.31* | 0.51* | 0.29* | 0.34* | 0.24 | 0.35* | 0.24* | 0.40* | 0.19* | 0.21* | 0.25* | 0.23* | 0.38* | 0.35* | 0.58* | 0.21* | 0.23* | 0.11* | 0.08* | 0.19* |  |  |  |  |  |  |
| KC | 0.39* | 0.32* | 0.43* | 0.37* | 0.30* | -0.03 | 0.06* | 0.41* | 0.21* | 0.16* | 0.22* | 0.22* | 0.13* | 0.11* | 0.11* | 0.01 | 0.02* | 0.27* | 0.25* | 0.27* | 0.47* | 0.01 | -0.01 | 0.10* | 0.07* | 0.00 | 0.20* |  |  |  |  |  |
| TLF | 0.39* | 0.32* | 0.42* | 0.37* | 0.34* | 0.06* | 0.10* | 0.41* | 0.20* | 0.20* | 0.21* | 0.24* | 0.14* | 0.17* | 0.12* | 0.08* | 0.10* | 0.27* | 0.26* | 0.28* | 0.49* | 0.00 | 0.04* | 0.04* | 0.05* | 0.02* | 0.15* | 0.03* |  |  |  |  |
| KT | 0.42* | 0.36* | 0.44* | 0.39* | 0.35* | 0.15* | 0.15* | 0.43* | 0.24* | 0.22* | 0.25* | 0.22* | 0.18* | 0.23* | 0.18* | 0.19* | 0.18* | 0.33* | 0.30* | 0.32* | 0.52* | 0.06* | 0.08* | 0.14* | 0.16* | 0.11* | 0.35* | 0.09* | 0.07* |  |  |  |
| TAC | 0.40* | 0.32* | 0.43* | 0.37* | 0.34* | 0.10* | 0.14* | 0.43* | 0.21* | 0.21* | 0.20* | 0.24* | 0.16* | 0.22* | 0.12* | 0.08* | 0.11* | 0.24* | 0.27* | 0.28* | 0.52* | 0.04 | 0.05* | 0.03 | 0.00 | 0.04* | 0.13* | 0.03* | 0.00* | 0.06* |  |  |
| HTB | 0.38* | 0.31* | 0.41* | 0.35* | 0.34* | 0.07* | 0.13* | 0.41* | 0.17* | 0.20* | 0.18* | 0.24* | 0.13* | 0.20* | 0.09* | 0.05* | 0.10* | 0.22* | 0.24* | 0.27* | 0.51* | 0.02 | 0.06 | 0.05 | 0.02 | 0.04* | 0.12* | 0.04* | 0.00* | 0.13* | 0.02* |  |
| SW | 0.39* | 0.32* | 0.43* | 0.36* | 0.31* | 0.07* | 0.07* | 0.42* | 0.19* | 0.16* | 0.19* | 0.21* | 0.12* | 0.13* | 0.09* | 0.05 | 0.04* | 0.25* | 0.25* | 0.26* | 0.51* | 0.00 | 0.00 | 0.09* | 0.05* | 0.02 | 0.23* | 0.00* | -0.01* | 0.03* | -0. 01* | 0.01* |

Population (Pop.) codes are given in Table 2. ^*^*P* < 0.05: significance level.

**Supplementary Table S6 Population pairwise *F*_ST_ values based on the combined mtDNA *COI* and *Cytb* sequences between the populations of *Aphis gosypii***

| Pop | CC | BC | LY | KZ | CX | LF | WR | DL | WQ | TC | TA | WF | JN | HZ | CD | MAS | CZ | NT | JJ | NC | JY | KEL | AKS | KS | JH | HM | SHZ | KC | TLF | KT | TAC | HTB |
| --- | --- | --- | --- | --- | --- | --- | --- | --- | --- | --- | --- | --- | --- | --- | --- | --- | --- | --- | --- | --- | --- | --- | --- | --- | --- | --- | --- | --- | --- | --- | --- | --- |
| CC |  |  |  |  |  |  |  |  |  |  |  |  |  |  |  |  |  |  |  |  |  |  |  |  |  |  |  |  |  |  |  |  |
| BC | -0.01 |  |  |  |  |  |  |  |  |  |  |  |  |  |  |  |  |  |  |  |  |  |  |  |  |  |  |  |  |  |  |  |
| LY | 0.93* | 1.00* |  |  |  |  |  |  |  |  |  |  |  |  |  |  |  |  |  |  |  |  |  |  |  |  |  |  |  |  |  |  |
| KZ | 0.14* | 0.16* | 0.82* |  |  |  |  |  |  |  |  |  |  |  |  |  |  |  |  |  |  |  |  |  |  |  |  |  |  |  |  |  |
| CX | 0.07* | 0.09 | 0.89* | 0.00 |  |  |  |  |  |  |  |  |  |  |  |  |  |  |  |  |  |  |  |  |  |  |  |  |  |  |  |  |
| LF | 0.37* | 0.45 | 0.89* | -0.01 | 0.13 |  |  |  |  |  |  |  |  |  |  |  |  |  |  |  |  |  |  |  |  |  |  |  |  |  |  |  |
| WR | 0.04 | 0.04 | 0.85* | 0.00 | -0.03 | 0.14 |  |  |  |  |  |  |  |  |  |  |  |  |  |  |  |  |  |  |  |  |  |  |  |  |  |  |
| DL | 0.04* | 0.05 | 0.87* | 0.03 | 0.00 | 0.17* | -0.01 |  |  |  |  |  |  |  |  |  |  |  |  |  |  |  |  |  |  |  |  |  |  |  |  |  |
| WQ | 0.18* | 0.21 | 0.83* | -0.04 | 0.01 | -0.03 | 0.03 | 0.06 |  |  |  |  |  |  |  |  |  |  |  |  |  |  |  |  |  |  |  |  |  |  |  |  |
| TC | 0.08* | 0.09* | 0.78* | -0.03 | -0.01 | 0.01 | 0.00 | 0.01 | -0.02 |  |  |  |  |  |  |  |  |  |  |  |  |  |  |  |  |  |  |  |  |  |  |  |
| TA | 0.16* | 0.18* | 0.67* | 0.07 | 0.10* | 0.13 | 0.10* | 0.09* | 0.08 | 0.06 |  |  |  |  |  |  |  |  |  |  |  |  |  |  |  |  |  |  |  |  |  |  |
| WF | 0.10* | 0.10* | 0.79* | -0.03 | -0.02 | 0.01 | 0.00 | 0.01 | -0.02 | -0.02 | 0.07 |  |  |  |  |  |  |  |  |  |  |  |  |  |  |  |  |  |  |  |  |  |
| JN | 0.02 | 0.02 | 0.81* | 0.05 | 0.01 | 0.19* | 0.01 | -0.03 | 0.08* | 0.03 | 0.04 | 0.03 |  |  |  |  |  |  |  |  |  |  |  |  |  |  |  |  |  |  |  |  |
| HZ | 0.19* | 0.19* | 0.78* | -0.03 | 0.03 | -0.04 | 0.05 | 0.07* | -0.04 | -0.01 | 0.10* | -0.01 | 0.10* |  |  |  |  |  |  |  |  |  |  |  |  |  |  |  |  |  |  |  |
| CD | 0.10 * | 0.11* | 0.78* | -0.01 | 0.01 | 0.04 | 0.02 | -0.01 | -0.02 | -0.01 | 0.05 | -0.01 | 0.02 | 0.00 |  |  |  |  |  |  |  |  |  |  |  |  |  |  |  |  |  |  |
| MAS | 0.00 | 0.00 | 0.89* | 0.06 | 0.01 | 0.24* | 0.00 | -0.03 | 0.10 | 0.03 | 0.11* | 0.04 | -0.02 | 0.11* | 0.03 |  |  |  |  |  |  |  |  |  |  |  |  |  |  |  |  |  |
| CZ | 0.24* | 0.26* | 0.78* | 0.00 | 0.09 | -0.05 | 0.09* | 0.12* | -0.01 | 0.02 | 0.12* | 0.02 | 0.14* | -0.01 | 0.04 | 0.16* |  |  |  |  |  |  |  |  |  |  |  |  |  |  |  |  |
| NT | 0.12* | 0.13* | 0.79* | 0.14* | 0.14* | 0.25* | 0.13* | 0.03 | 0.18* | 0.11* | 0.15* | 0.13* | 0.05 | 0.20* | 0.06 | 0.06* | 0.22* |  |  |  |  |  |  |  |  |  |  |  |  |  |  |  |
| JJ | 0.06* | 0.07* | 0.78* | 0.00 | 0.01 | 0.07 | 0.01 | -0.03 | 0.03 | 0.00 | 0.06 | 0.01 | -0.01 | 0.05 | -0.02 | 0.00 | 0.06 | 0.01 |  |  |  |  |  |  |  |  |  |  |  |  |  |  |
| NC | 0.05* | 0.04* | 0.76* | 0.01 | 0.00 | 0.08 | -0.01 | 0.01 | 0.02 | 0.00 | 0.07* | 0.00 | 0.02 | 0.04 | 0.01 | 0.01 | 0.05 | 0.10* | 0.01 |  |  |  |  |  |  |  |  |  |  |  |  |  |
| JY | 0.65* | 0.63* | 0.74* | 0.59* | 0.65* | 0.59* | 0.65* | 0.62* | 0.61* | 0.59* | 0.58* | 0.61* | 0.64* | 0.64* | 0.60* | 0.62* | 0.61* | 0.61* | 0.57* | 0.62* |  |  |  |  |  |  |  |  |  |  |  |  |
| KEL | 0.00 | -0.02 | 0.94* | 0.16* | 0.09 | 0.41* | 0.05 | 0.05* | 0.21* | 0.10* | 0.19* | 0.11* | 0.03* | 0.21* | 0.12* | 0.01 | 0.27* | 0.14* | 0.08* | 0.06* | 0.67* |  |  |  |  |  |  |  |  |  |  |  |
| AKS | 0.01 | 0.00 | 1.00* | 0.21* | 0.12* | 0.52* | 0.06 | 0.07* | 0.26* | 0.12* | 0.23* | 0.14* | 0.04* | 0.23* | 0.14* | 0.02 | 0.31* | 0.16* | 0.10* | 0.06* | 0.67* | 0.00 |  |  |  |  |  |  |  |  |  |  |
| KS | 0.01 | 0.00 | 0.83* | 0.05 | 0.00 | 0.19* | -0.01 | 0.00 | 0.08* | 0.02 | 0.10* | 0.03 | 0.00 | 0.10* | 0.04* | -0.01 | 0.14* | 0.10* | 0.02 | 0.01 | 0.64* | 0.01 | 0.01 |  |  |  |  |  |  |  |  |  |
| JH | 0.06 | 0.09 | 0.91* | 0.00 | -0.04 | 0.14 | -0.03 | -0.01 | 0.02 | -0.02 | 0.10* | -0.02 | 0.00 | 0.04 | 0.00 | 0.00 | 0.09* | 0.13* | 0.01 | -0.01 | 0.63* | 0.07* | 0.12 | -0.01 |  |  |  |  |  |  |  |  |
| HM | 0.01 | 0.01 | 0.94* | 0.11* | 0.01 | 0.35* | 0.00 | 0.01 | 0.14* | 0.06 | 0.18* | 0.06* | 0.01* | 0.15* | 0.08* | -0.01 | 0.22* | 0.15* | 0.06 | 0.03 | 0.68* | 0.01 | 0.02 | -0.01 | 0.00 |  |  |  |  |  |  |  |
| SHZ | 0.02 | 0.04 | 0.91* | 0.01 | -0.02 | 0.16 | -0.03 | -0.02 | 0.04 | -0.01 | 0.06 | 0.00 | -0.02 | 0.06 | 0.00 | -0.02 | 0.10 | 0.08 | -0.01 | -0.02 | 0.58* | 0.03 | 0.08 | -0.03 | -0.03 | -0.01 |  |  |  |  |  |  |
| KC | 0.02 | 0.00 | 1.00* | 0.25* | 0.15* | 0.58* | 0.08* | 0.10* | 0.30* | 0.15* | 0.27* | 0.16* | 0.05* | 0.26* | 0.17* | 0.03* | 0.35* | 0.19* | 0.13 | 0.08* | 0.70* | 0.01 | 0.00 | 0.02 | 0.15* | 0.03 | 0.11 |  |  |  |  |  |
| TLF | 0.24* | 0.27* | 0.85* | -0.03 | 0.04 | -0.05 | 0.06 | 0.09 | -0.04 | -0.01 | 0.11* | -0.02 | 0.11* | -0.04 | 0.01 | 0.14* | -0.02 | 0.21* | 0.05* | 0.04 | 0.62* | 0.27* | 0.32* | 0.11* | 0.05 | 0.20* | 0.08 | 0.37* |  |  |  |  |
| KT | -0.04 | 0.00 | 1.00* | 0.09 | 0.05 | 0.35* | 0.01 | 0.01 | 0.15* | 0.04 | 0.11* | 0.05 | -0.01 | 0.15 | 0.05 | -0.03 | 0.19* | 0.08 | 0.01 | 0.01 | 0.58* | -0.04 | 0.00 | -0.03 | 0.04 | -0.02 | -0.01 | 0.00 | 0.21 |  |  |  |
| TAC | -0.05 | 0.00 | 1.00* | 0.07 | 0.03 | 0.32 | -0.01 | -0.01 | 0.13 | 0.02 | 0.09 | 0.04 | -0.03 | 0.13 | 0.04 | -0.05 | 0.17* | 0.06 | 0.00 | -0.01 | 0.57* | -0.05 | 0.00 | -0.05 | 0.03 | -0.03 | -0.03 | 0.00 | 0.19 | 0.00 |  |  |
| HTB | -0.09 | 0.00 | 1.00* | 0.03 | 0.00 | 0.26 | -0.04 | -0.05 | 0.09 | -0.02 | 0.04 | 0.00 | -0.06 | 0.09 | 0.00 | -0.08 | 0.13 | 0.02 | -0.04 | -0.04 | 0.54* | -0.09 | 0.00 | -0.08 | -0.01 | -0.07 | -0.07 | 0.00 | 0.15 | 0.00 | 0.00 |  |
| SW | 0.01 | 0.10 | 0.95* | -0.03 | -0.07 | 0.12 | -0.07 | -0.05 | 0.00 | -0.05 | 0.04 | -0.05 | -0.04 | 0.01 | -0.03 | -0.05 | 0.05 | 0.07 | -0.04 | -0.05 | 0.56* | 0.03 | 0.15 | -0.06 | -0.08 | -0.05 | -0.08 | 0.20 | 0.03 | 0.02 | 0.00 | -0.05 |

Population (Pop.) codes are given in Table 2. ^*^*P* < 0.05: significance level.

**Supplementary Table S7** Wilcoxon signed rank test for mutation–drift equilibrium estimated based on 10 microsatellite loci

| Pop. | IAM | TPM | SMM. | Mode shift |
| --- | --- | --- | --- | --- |
| CC | 0.920 | 0.984 | 0.999 | L |
| BC | 0.884 | 0.988 | 0.998 | L |
| LY | 0.958 | 0.999 | 1.000 | L |
| KZ | 0.410 | 0.850 | 0.936 | L |
| CX | 0.348 | 0.984 | 0.999 | L |
| LF | 0.752 | 0.976 | 0.997 | L |
| WR | 0.500 | 0.884 | 0.997 | L |
| DL | 0.012 | 0.246 | 0.935 | L |
| WQ | 0.722 | 0.935 | 0.999 | L |
| TC | 0.138 | 0.722 | 0.991 | L |
| TA | 0.213 | 0.455 | 0.875 | L |
| WF | 0.285 | 0.787 | 0.993 | L |
| JN | 0.278 | 0.884 | 0.997 | L |
| HZ | 0.711 | 0.980 | 0.992 | L |
| CD | 0.590 | 0.898 | 0.998 | L |
| MAS | 0.371 | 0.371 | 0.973 | L |
| CZ | 0.898 | 0.993 | 0.998 | L |
| NT | 0.285 | 0.936 | 0.997 | L |
| JJ | 0.754 | 0.839 | 0.991 | L |
| NC | 0.188 | 0.615 | 0.988 | L |
| JY | 0.273 | 0.727 | 0.990 | L |
| KEL | 0.006 | 0.473 | 0.996 | L |
| AKS | 0.248 | 0.820 | 0.981 | L |
| KS | 0.125 | 0.752 | 0.997 | L |
| JH | 0.125 | 0.545 | 0.990 | L |
| HM | 0.027 | 0.902 | 0.998 | L |
| SHZ | 0.539 | 0.935 | 0.997 | L |
| KC | 0.422 | 0.770 | 0.994 | L |
| TLF | 0.010 | 0.589 | 0.993 | L |
| KT | 0.002 | 0.037 | 0.578 | L |
| TAC | 0.323 | 0.680 | 0.999 | L |
| HTB | 0.002^**^ | 0.002^**^ | 0.004^**^ | S |
| SW | 0.002^**^ | 0.010^**^ | 0.191 | S |
| Eastern region group | 0.652 | 1.000 | 1.000 | L |
| Western region group | 0.784 | 1.000 | 1.000 | L |
| Total region | 0.920 | 1.000 | 1.000 | L |

*P* is test for heterozygosity excess, ^**^*P* < 0.01; Pop.: population; IAM: infinite allele model; TPM: two-phase model; SMM: stepwise mutation model; L: normal L-shaped distribution; S: shifted mode.
